# Genomic analysis reveals deep population divergence in the water snake *Trimerodytes percarinatus* (Serpentes, Natricidae)

**DOI:** 10.1002/ece3.11278

**Published:** 2024-04-15

**Authors:** Bing Lyu, Qin Liu, Yayong Wu, Truong Q. Nguyen, Jing Che, Sang N. Nguyen, Edward A. Myers, Frank T. Burbrink, Peng Guo, Jichao Wang

**Affiliations:** ^1^ Ministry of Education Key Laboratory for Ecology of Tropical Islands, Key Laboratory of Tropical Animal and Plant Ecology of Hainan Province, College of Life Sciences Hainan Normal University Haikou China; ^2^ Faculty of Agriculture, Forestry and Food Engineering Yibin University Yibin China; ^3^ Institute of Ecology and Biological Resources Vietnam Academy of Science and Technology Hanoi Vietnam; ^4^ Vietnam Academy of Science and Technology Graduate University of Science and Technology Hanoi Vietnam; ^5^ State Key Laboratory of Genetic Resources and Evolution & Yunnan Key Laboratory of Biodiversity and Ecological Conservation of Gaoligong Mountain, Kunming Institute of Zoology Chinese Academy of Sciences Kunming China; ^6^ Institute of Tropical Biology Vietnam Academy of Science and Technology Ho Chi Minh City Vietnam; ^7^ Department of Herpetology California Academy of Sciences San Francisco California USA; ^8^ Department of Herpetology American Museum of Natural History New York New York USA

**Keywords:** Asia, genetic diversity, population genetics, snake

## Abstract

Although several phylogeographic studies of Asian snakes have been conducted, most have focused on pitvipers, with non‐venomous snakes, such as colubrids or natricids, remaining poorly studied. The Chinese keelback water snake (*Trimerodytes percarinatus* Boulenger) is a widespread, semiaquatic, non‐venomous species occurring in China and southeastern Asia. Based on mitochondrial DNA (mtDNA) and single nucleotide polymorphism (SNP) data, we explored the population genetic structure, genetic diversity, and evolutionary history of this species. MtDNA‐based phylogenetic analysis showed that *T. percarinatus* was composed of five highly supported and geographically structured lineages. SNP‐based phylogenetic analysis, principal component analysis, and population structure analysis consistently revealed four distinct, geographically non‐overlapping lineages, which was different from the mtDNA‐based analysis in topology. Estimation of divergence dates and ancestral area of origin suggest that *T. percarinatus* originated ~12.68 million years ago (95% highest posterior density: 10.36–15.96 Mya) in a region covering southwestern China and Vietnam. Intraspecific divergence may have been triggered by the Qinghai‐Xizang Plateau uplift. Population demographics and ecological niche modeling indicated that the effective population size fluctuated during 0.5 Mya and 0.002 Mya. Based on the data collected here, we also comment on the intraspecific taxonomy of *T. percarinatus* and question the validity of the subspecies *T. p. suriki*.

## Graphical abstract

In this work, we used both mtDNA data and SNP data to explore the population genetic structure, genetic diversity, and evolutionary history of a widespread, semiaquatic, non‐venomous snake *Trimerodytes percarinatus*. We hope our present study will be of wide interest to systematic researchers and phylogeographers.

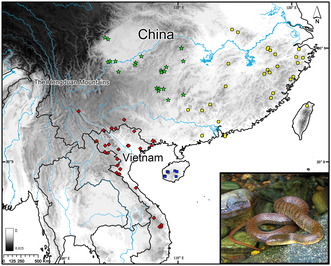

## 1 INTRODUCTION

The patterns of genetic diversity, population structure, and evolutionary history of organisms have been greatly affected by various biological factors and abiotic factors. Of those, geography and climatic oscillations have been previously shown to generate population structure, drive the process of speciation, and influence population size contraction and expansion (Burbrink et al., 2016, 2022; Hewitt, 2004; Myers et al., 2017; Qu & Lei, 2009; Yang et al., 2022). Complex geological events can result in physical barriers, such as mountains, rivers, and marine incursions, which can prevent gene flow between closely related species or populations. Furthermore, unfavorable climate changes should cause populations to contract, on the contrary, a favorable climate may lead to population expansion (Berv et al., 2021; Fijarczyk et al., 2011; Li et al., 2022; Myers et al., 2019, 2020; Pyron & Burbrink, 2009; Ursenbacher et al., 2008; Yang et al., 2022; Zhao et al., 2022). However, the influence of both factors on these processes differ in the timing and effect among different regions (such as Asia and North America) and among groups (such as birds and reptiles).

East and Southeast Asia contain four global biodiversity hotspots (i.e., Himalaya region, mountains of Southwest China, Indo‐Burma, and Sundaland; Mittermeier et al., 2011). The biodiversity of this region has been heavily studied with regard to taxonomy, evolution, biogeography, and population genetics (Che et al., 2010; Guo et al., 2011, 2016, 2019, 2020; Hofmann et al., 2015; Wu et al., 2020; Yan et al., 2013; Zhu et al., 2016, 2022). A large number of these studies have focused on the evolutionary history of species within this region and speciation patterns have been assessed among various taxa during the last two decades, including fish (Zhao et al., 2005), amphibians (Lin et al., 2023; Murphy et al., 2000; Wu et al., 2020; Zhang et al., 2008), birds (Chen et al., 2015), and mammals (Li et al., 2005; Pang et al., 2003; Patou et al., 2010). However, research on snakes, which are highly sensitive to climatic fluctuations and exhibit constrained dispersal capabilities, remains limited and heavily focused on venomous taxa (Ding et al., 2011; Guo et al., 2011, 2012, 2016, 2019; Huang et al., 2007; Lin et al., 2014; Zhu et al., 2022).

The Chinese keelback water snake, *Trimerodytes percarinatus*, is a semiaquatic, nonvenomous colubrid with a wide geographic distribution in China and Southeast Asia (Das, 2010; Uetz et al., 2023; Zhao, 2006). It is primarily nocturnal and can be found in various waterbodies, such as streams, rivers, and ponds, at elevation ranging from 100 to 2000 meters above sea level (Zhao, 2006). Currently, two subspecies are generally recognized, *Trimerodytes percarinatus percarinatus* (Boulenger, 1899), based on type specimens from Fujian, China, and *Trimerodytes percarinatus suriki* (Maki, 1931), based on specimens from Taiwan, China (Zhao et al., 1998). The two subspecies differ from each other only in terms of the number of ventral scales (Zhao et al., 1998). However, Zhao (2006) questioned the validity of *T. p. suriki* and suggested that further research was needed to evaluate its taxonomic status.

The widespread geographic distribution and specialized ecology make *T. percarinatus* a good model for evaluating the impact of geographical features and climatic cycles on the demographic history of species in East and Southeast Asia. Herein, based on extensive geographic sampling, integration of mitochondrial DNA (mtDNA) and genome‐wide single nucleotide polymorphism (SNP) markers, we explored the population structure and evolutionary history of this species. We aimed to (1) determine the population structure and evaluate genetic diversity and (2) establish the timing of species diversification and evolutionary dynamics within the species.

## 2 MATERIALS AND METHODS

### 2.1 Taxon sampling and DNA extraction

**FIGURE 1:**
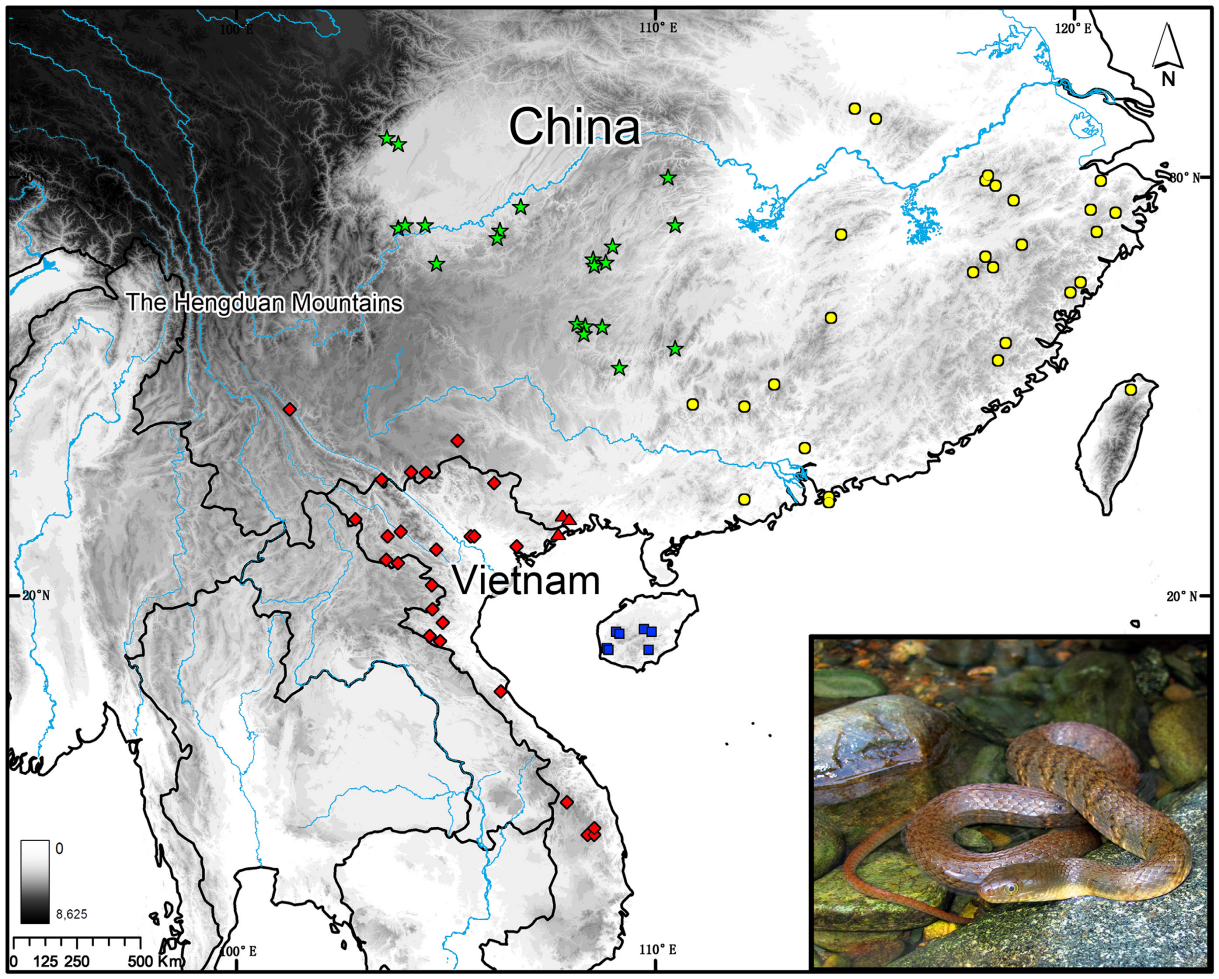
Map showing localities of *Trimerodytes percarinatus* sampled in this study. Symbols indicate different mtDNA lineages and colors represent SNP‐based lineages. Square: A, Triangle: B, Circle: C, Pentagram: D, Diamond: E. Blue: I, Red: II, Yellow: III, Green: IV.

Samples were obtained via fieldwork or provided from colleagues and natural history museums. In total, 180 samples of *T. percarinatus* from 71 localities covering almost its entire geographic range were collected, sequenced, and analyzed (Figure 1; Table S1). In addition, five samples from known congeners *T. annularis*, *T. balteatus*, *T. aequifasciatus*, *T. yunnanensis*, and *T. yapingi* were included, with *T. yapingi* chosen as the outgroup based on a previous phylogenetic study (Guo et al., 2020). Total genomic DNA was isolated from liver, muscle, or skin tissue preserved in 85% ethanol using an Animal Genomic DNA Purification Kit (Tiangen Bio‐Tech Co., Ltd., Beijing, China).

### 2.2 Mitochondrial DNA sequencing and SNP generation

Two mitochondrial gene fragments, cytochrome b (*cyt.b*) and NADH dehydrogenase subunit 2 (*ND2*), were amplified with the primers L14910/H16064 (Burbrink et al., 2000) and hxND2F/hxND2R (Reynolds, 2011), respectively. The polymerase chain reaction (PCR) parameters were identical to those described in the corresponding references for each primer pair. The PCR products were purified, with the double‐stranded products bidirectionally sequenced at a commercial sequencing company (Sangon, Chengdu, China).

Based on the mtDNA phylogenetic analysis (see below), 61 individuals representing all mtDNA lineages were selected to generate restriction site‐associated DNA sequencing (RAD‐seq) libraries following the protocols of Peterson et al. (2012) and Schield et al. (2015). In brief, DNA was digested with the restriction enzyme *Eco*RI, and unique barcode adapters were ligated onto each individual sample in the library. Sequencing of 200–400 bp fragments was performed on a single lane of an Illumina HiSeq sequencer using a paired‐end protocol by Novogene Ltd. (Tianjin, China).

The raw RAD‐seq Illumina reads were subjected to quality control and filtered using the Novogene QC program (parameter: ‐r 150 ‐N 0.1 ‐q 33 ‐L 5 ‐p 0.5) to obtain reliable reads and avoid those with artificial biases. The following types of reads were removed during quality control: (i) those containing ≥10% unidentified nucleotides; (ii) those with more than 10 nucleotides aligned to the adaptor or mismatches exceeding 10%; (iii) those with over 50% of read bases with Phred quality scores lower than 5; and (iv) putative PCR duplicates generated during library construction. The RAD‐seq data were assembled using SOAPdenovo2 (Luo et al., 2012) with the parameters (max_rd_len = 150, avg_ins = 200, reverse_seq = 0, asm_flags = 3, rd_len_cutoff = 150, rank = 1‐K 47) to generate contigs. The contigs were aligned to the pseudo‐reference sequence by using BWA (Li & Durbin, 2009) with the command “mem ‐t 4 ‐k 32 ‐M” to generate SAM files, which were imported into SAMtools (Li et al., 2009) for sorting and merging, and then converted into BAM files. Simultaneously, both sequencing depth and coverage were calculated according to the BWA alignment results. Finally, BAM files were imported into SAMtools (Li et al., 2009) for SNP detection. The minimum percentage of individuals in a population required to retain a locus for that population (‐r) was set to 80%. All SNPs with a minor allele frequency (maf) < 0.05 were excluded from the genotype dataset of all samples.

### 2.3 Phylogenetic analyses

All mtDNA sequences were assembled and edited manually using SeqMan in Lasergene v15.1 (DNASTAR, USA). Sequences were aligned using the MUSCLE algorithm implemented in MEGA v7 (Kumar et al., 2016), and both segments were concatenated in the software MEGA v7 (Kumar et al., 2016). Sequences were converted into amino acid residues to check for stop codons and ensure an open reading frame. PartitionFinder v2.1 (Lanfear et al., 2017) was used to identify the best partitioning schemes and model of sequence evolution for each partition based on the Akaike information criterion (AIC). Bayesian inference (BI) and Maximum likelihood (ML) methods were used to infer phylogenetic relationships. Bayesian analyses were executed using MrBayes v3.2.2 (Ronquist et al., 2012). All searches were performed with three independent runs, each initiating from a random tree. Each run consisted of four Markov chains (three heated and one cold chain) for 2 × 10^7^ generations, sampled every 2000 generations, with the first 25% discarded as burn‐in. Effective sample size (ESS) was determined using Tracer v1.7.2 (Rambaut et al., 2018) to ensure all parameter values had ESS > 200. ML analysis was performed using IQ‐TREE v2.0 (Minh et al., 2020) with the same partition strategy and evolutionary models as the BI analysis and nodal support were assessed using the ultrafast bootstrap approximation (UFBoot) with 1000 replicates (Hoang et al., 2018). Trees were visualized in FigTree v1.4.3 (Rambaut, 2014), nodes with posterior probability (PP) ≥0.95 and UFBoot >95% were considered as well‐supported (Hoang et al., 2018; Huelsenbeck & Rannala, 2004).

Phylogenetic analyses were conducted for the SNP dataset using a ML approach with the GTRGAMMA model and outgroup *T. yapingi*. The ML analysis was performed in RAxML v8.2.11 (Stamatakis, 2006), with branch support assessed by 100 rapid bootstrap replicates.

### 2.4 Genetic diversity and population structure

The observed heterozygosity (*Ho*), expected heterozygosity (*He*), and the average inbreeding coefficient (*Fis*) in each lineage of the SNP‐based phylogenetic tree were calculated in Hierfstat v0.5–11 (Goudet, 2005) with default parameters, and *Fis* was calculated with bootstrapping the loci (n = 1000, confidence interval: 0.025–0.975). Nucleotide diversity (π) was estimated in Arlequin v 3.5 (Excoffier & Lischer, 2010) and the differentiation coefficient (*Fst*) among SNP‐based lineages was calculated in VCFtools v 0.1.16 (Danecek et al., 2011), where low values indicate little population differentiation. The mean uncorrected *p* distances of each SNP‐based lineage were calculated in MEGA v7 (Kumar et al., 2016). Several mtDNA‐based genetic diversity indices, including the number of haplotypes (*H*), haplotype diversity (*Hd*), and nucleotide diversity (π), were also computed in DnaSP v 5.10 (Librado & Rozas, 2009) and Arlequin v 3.5 (Excoffier & Lischer, 2010), and the average pairwise distances (*p*‐distances) among clades based on mtDNA gene tree were calculated in MEGA v7 (Kumar et al., 2016).

To assess the number of genetic groups within *T. percarinatus*, we used multivariate and model‐based methods for the SNP data. First, a principal component analysis (PCA) was conducted in Plink v1.9 (Chang et al., 2015), and the first three principal components were plotted. Second, population structure was inferred assuming different numbers of clusters (K) from 2 to 8 using Admixture v1.3.0 (Alexander et al., 2009). We used 10‐fold cross‐validation (CV) to compare different numbers of clusters, with the lowest CV value indicating the most likely number of clusters.

### 2.5 Gene flow analyses

To investigate gene flow among the SNP‐based lineages, two different methods were used. First, the D and f4 statistics (from the ABBA‐BABA test; Green et al., 2010) were used to estimate intraspecific gene flow between all of the *T. percarinatus* lineages based on SNP data using “qpDstat” in AdmixTools (Patterson et al., 2012), *T. yapingi* was also included as the outgroup. The statistical significance of D and f4 statistics was assessed through a block‐jackknifing approach as implemented in AdmixTools, in which the absolute value of a Z‐score > 3 was considered significant (Patterson et al., 2012). Second, TreeMix v 1.13 (Pickrell & Pritchard, 2012), which applies an ML method based on a Gaussian model of allele frequency change, was conducted using 5 different migration parameter settings, ranging from zero to 4 migration events. For each migration setting, we conducted 1000 bootstrap replicate analyses and identified the highest likelihood run from this set of 1000 to characterize evidence for the best‐fit migration model.

### 2.6 Divergence date and ancestral area estimation

BEAST v 2.6.6 (Bouckaert et al., 2014) was used to estimate the date of origin of *T. percarinatus* as well as each lineage based on all mtDNA sequences using a Yule model and a relaxed uncorrelated lognormal clock (Drummond et al., 2006). The following three calibration constraints were used: (1) The most recent common ancestor (MRCA) of all Natricinae was constrained with a lognormal mean of 30 Mya and standard deviation (SD) of 0.115, yielding a 95% confidence interval (CI) from 23 to 36 Mya; (2) MRCA of *Natrix* was constrained with a lognormal mean of 22 Mya and SD of 0.15, yielding a 95% CI of 16–28 Mya; (3) MRCA of the genus *Thamnophis* was constrained with a lognormal mean of 16 Mya and SD of 0.15, yielding a 95% CI interval of 12–20 Mya (Guo et al., 2012). For these calibrations, several additional sequences were retrieved from GenBank, with *Acrochordus granulatus* chosen as the outgroup (Table S1). Two independent searches were conducted based on 2 × 10^7^ generations and sampling every 1000 iterations, with the first 25% of samples discarded as burn‐in. Both analyses produced similar results and yielded ESS values >200 for the posterior probability distribution of all parameters (Drummond et al., 2006), as calculated in Tracer v 1.7 (Rambaut et al., 2018).

To infer the biogeographic history of *T. percarinatus*, we estimated ancestral area using dispersal vicariance analysis (S‐DIVA) and dispersal extinction cladogenesis (DEC) using the program Reconstruct Ancestral States in Phylogenies (RASP; Yu et al., 2014). Analyses were conducted for one million cycles, with 25% of the initial run discarded as burn‐in. The reconstructed biogeographic region for each node was summarized and plotted as a pie chart. Based on the geographic distributions of mtDNA lineages, distributions were coded as southwestern China (SWC), central China (CC), southeastern China (SEC), and southern China and Vietnam (SCV) (Shi et al., 2006; Zhao et al., 2005; Zhu, 2016).

### 2.7 Demographic history

To determine changes in population size over time, we examined the historical demographics within *T. percarinatus* lineages using Multiple Sequentially Markovian Coalescent (MSMC2, Schiffels & Wang, 2020) based on the SNP dataset. First, the program bcftools (Li, 2011) was used to filter out the sites with missing genotype “. /.” from each lineage of the SNP‐based phylogenetic tree, and two samples were randomly selected for each lineage. Then, the MSMC2 analysis was executed following Dong et al. (2023) with the parameters as follows: (1) the generation time (g) was set to 3 years; (2) the generation mutation rate (μ) was 4.71 × 10^−9^ per site per generation (Peng et al., 2020).

### 2.8 Ecological niche modeling

To evaluate the influences of glacial cycles on the geographic distribution of *T. percarinatus*, we predicted suitable habitat during the Last Glacial Maximum, Mid‐Holocene, and at present using ecological niche modeling (ENM). Geographic coordinates were obtained from a variety of sources, including museum records of 535 *T. percarinatus* specimens from Vertnet (www.vertnet.org), published accounts (e.g., Yang & Rao, 2008; Zhao et al., 1998), and field surveys. After filtering duplicate locations that were within 5 km^2^ of one another, a total of 233 geographic coordinates were retained for *T. percarinatus*. The 19 bioclimatic variables for the present (1950–2000), Mid‐Holocene (~0.006 Mya), and the Last Glacial Maximum (LGM, 0.022–0.011 Mya) were downloaded from WorldClim v1.4 (http://www/worldclim.org) with a resolution of 2.5 arc minutes (Hijmans et al., 2005). We used a Pearson's correlation coefficient threshold of 0.85 to identify correlated variables. We retained 11 climatic variables (Table S2) after removing 8 climatic variables with high correlations. The ENM was conducted using Maxent v 3.4.4 (Phillips et al., 2006). We used 75% of coordinates as training data and 25% as testing data. Simulations were conducted for 10 replicates, and the average result was selected as the best model output. The area under the receiver operating characteristic curve (AUC) was used to evaluate simulation accuracy, with AUC values >0.9 indicating good performance. We used Arcgis v10.4 (ESRI) to visualize the ENMs at the LGM, Mid‐Holocene, and present day.

## 3 RESULTS

The final aligned dataset of mtDNA sequences consisted of 2081 bp (1065 bp from *cyt.b* and 1016 bp from *ND2*). Of these, 32 sequences were from our previous published work (Guo et al., 2020). All novel sequences generated were deposited in GenBank (accession numbers OQ575713–OQ576010; Table S1). Our RAD‐seq libraries returned 13.79–91.55 × 10^6^ reads per sample (mean = 18.46 × 10^6^), with a read length of 150 bp. After filtering for low‐quality reads, we retained 13.63–91.37 × 10^6^ reads per sample (mean = 18.24 × 10^6^). After assembling and filtering these genomic data, a total of 393,329 SNPs were obtained (mean = 6051 SNPs per sample) from all samples.

### 3.1 Phylogenetic analyses

**FIGURE 2:**
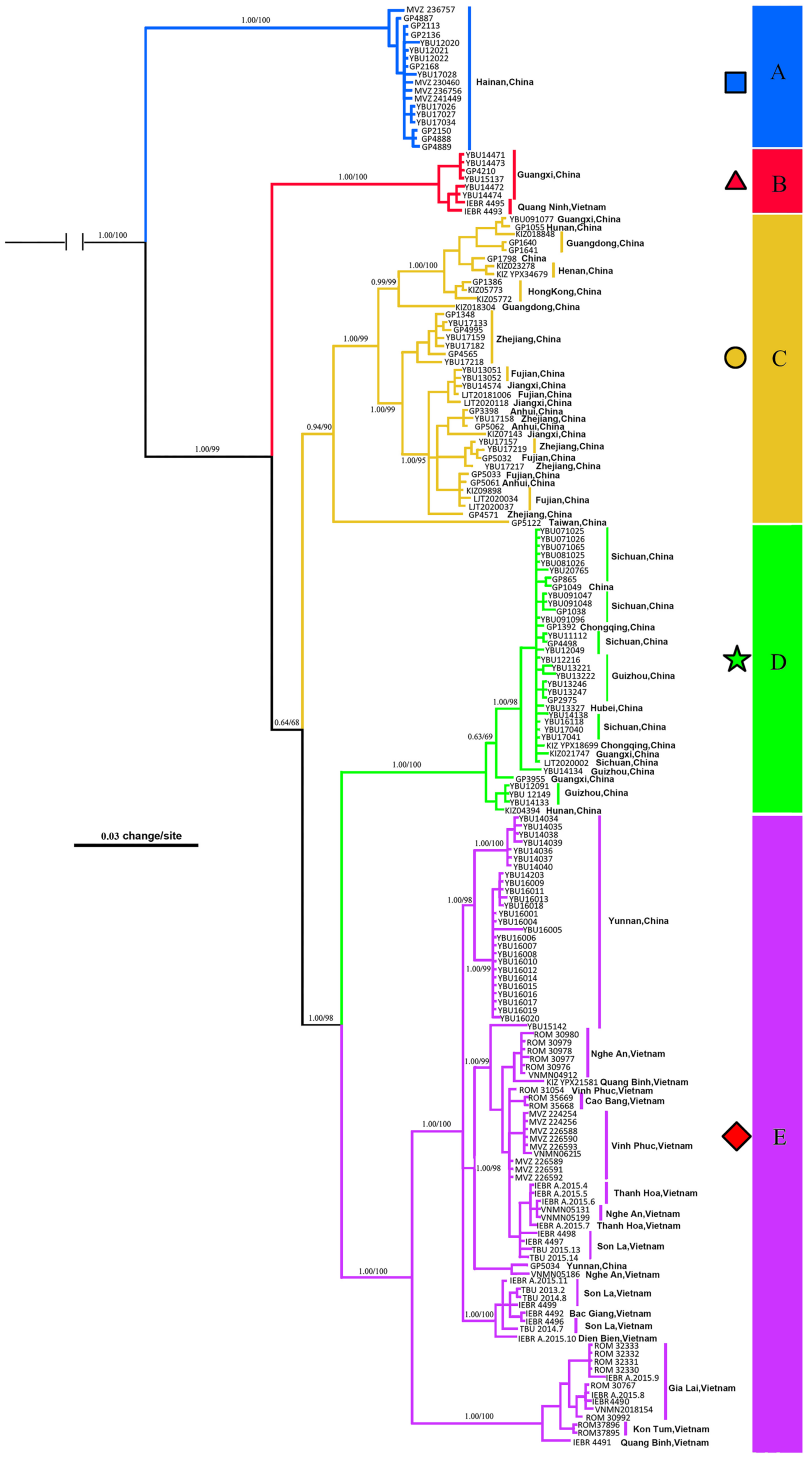
Bayesian 50% majority‐rule consensus tree of *Trimerodytes percarinatus* inferred from the mtDNA dataset. Posterior probabilities from BI (>50%) and bootstrap support values from ML analyses (>50) are adjacent to major nodes. Branch support indices are not given for most shallow nodes to preserve clarity. Square: A, Triangle: B, Circle: C, Pentagram: D, Diamond: E. Blue: I, Red: II, Yellow: III, Green: IV.

The mtDNA‐based BI and ML gene trees showed similar topologies, with slight disagreements at some shallow nodes (Figure 2). Analyses consistently indicated that all *T. percarinatus* samples formed a well‐supported monophyletic group (1.00 PP and 100% UFB) with five major lineages, each receiving high support values (A–E; Figures 1 and 2). Lineage A, which was sister to all other lineages, consisted of the specimens from Hainan Island exclusively. Lineage B was composed of specimens from southwestern Guangxi in China and northeastern Vietnam. Lineage C contained samples from eastern and central China, including Taiwan, Hong Kong, Guangdong, Fujian, Zhejiang, Jiangxi, Anhui, southern Henan, eastern Hunan, and northeastern Guangxi. One specimen from Taiwan was sister to all other samples in lineage C. Lineage D included specimens from southwestern China, including Sichuan, Chongqing, Guizhou, western Hubei, western Hunan, and northwestern Guangxi. Lineage E, sister to lineage D, contained samples from Yunnan in China and the rest of Vietnam, excluding northeastern Vietnam. Although lineages B–E formed highly supported groups, the relationships among B, C, and D + E were unresolved (Figures 1 and 2).

**FIGURE 3:**
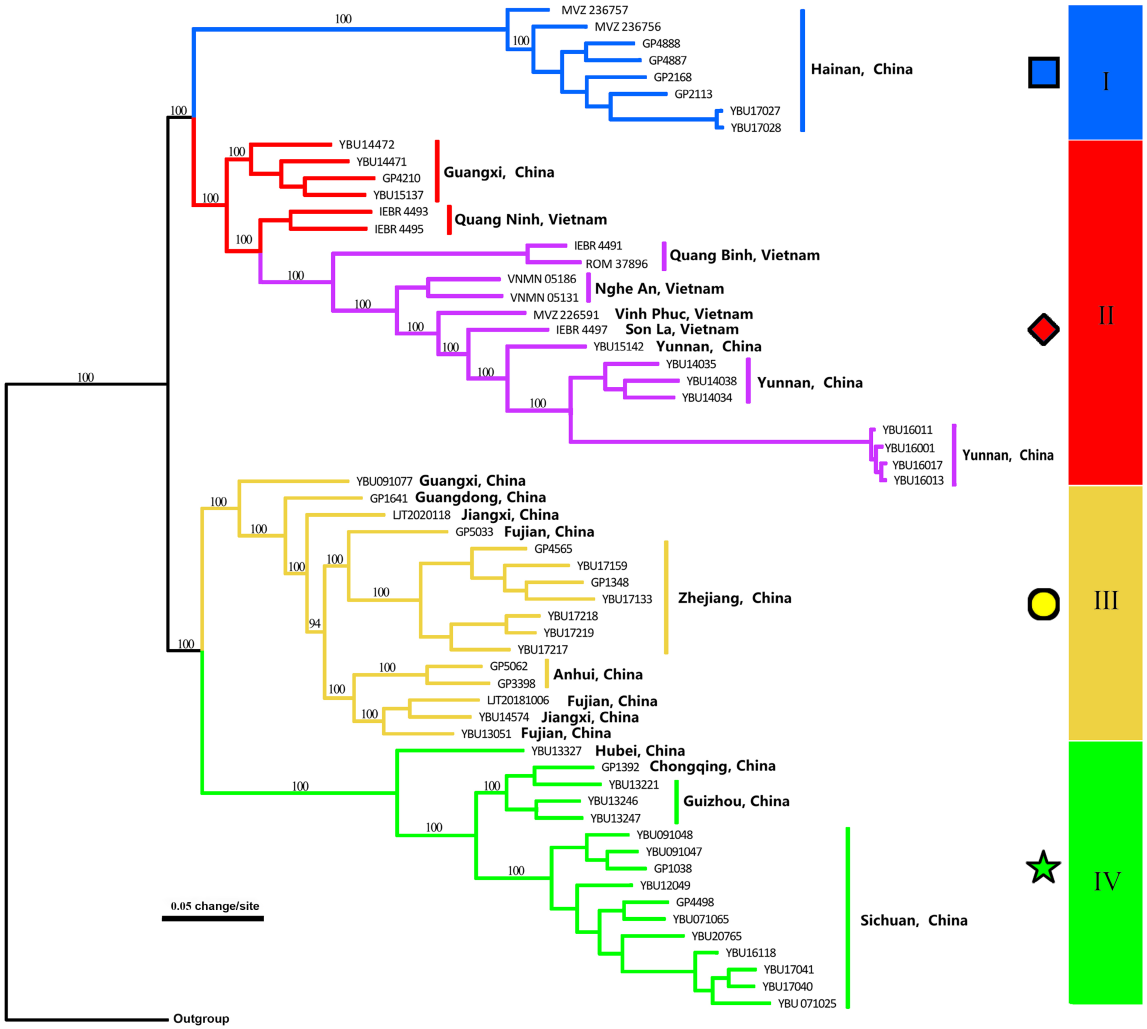
Maximum likelihood phylogeny inferred from genome‐wide SNP data. Numbers above branches indicate bootstrap values for each node. Square: A, Triangle: B, Circle: C, Pentagram: D, Diamond: E. Blue: I, Red: II, Yellow: III, Green: IV.

The SNP‐based phylogenetic relationships were generally consistent with those estimated from mtDNA, but differed in a few specific ways. The ML analyses indicated that all *T. percarinatus* samples formed a highly supported monophyletic group (BS 100%) with only four distinct and well‐supported lineages (I–IV; Figure 3). The samples from mtDNA lineages A, C, and D formed distinct and highly supported lineages labeled as I, III, and IV with the genomic data. While those samples from lineages B and E formed a monophyletic lineage II. It is likely that these lineages are parapatric. The interlineage relationships were different in both analyses, but well resolved in the genomic data tree.

### 3.2 Genetic diversity and population structure

Based on the SNP data, *Ho* ranged from 0.1103 (lineage IV) to 0.1587 (lineage I) with a mean of 0.1433 per lineage; *He* ranged from 0.1458 (lineage IV) to 0.2247 (lineage II), with an average of 0.1815. The estimated *Fis* ranged from 0.0289 (lineage I) to 0.3337 (lineage II). The highest nucleotide diversity was found in lineage II (0.0770) and the lowest in lineage IV (0.0041) (Table 1). The uncorrected interlineage genetic distances (*p*‐distances) ranged from 2.88% (III and IV) to 26.81% (I and II); Pairwise *Fst* values were all statistically significant, ranging from 0.1633 (II and III) to 0.3428 (I and IV) (Table 2).

**TABLE 1:** Genetic diversity of SNP data for each lineage.

Lineage	*N*	*Ho*	*He*	*Fis*	π
I	8	0.1587	0.1634	0.0289	0.0047
II	20	0.1497	0.2247	0.3337	0.0770
III	16	0.1545	0.1921	0.1955	0.0054
IV	16	0.1103	0.1458	0.2436	0.0041

Note: *Fis*, average inbreeding coefficient; *He*, expected heterozygosity; *Ho*, observed heterozygosity; N, number of individuals; π, nucleotide diversity.

**TABLE 2:** The mean uncorrected *p* distances and differentiation (*Fst*) between the four lineages based on SNP dataset.

Lineage	I	II	III	IV
I	—	0.1982	0.2296	0.3428
II	26.81%	—	0.1633	0.2252
III	22.73%	7.65%	—	0.2326
IV	25.39%	9.66%	2.88%	—

Note: Below the diagonal is the mean uncorrected‐p distances and above the diagonal is the differentiation (*Fst*).

The mean interlineage uncorrected *p* distances ranged from 3.39% to 6.02% based on *cyt.b* and from 2.77% to 4.86% based on *ND2* (Table S3). The overall *Hd* was 0.96, π was 0.029–0.031 (Table S4).

**FIGURE 4:**
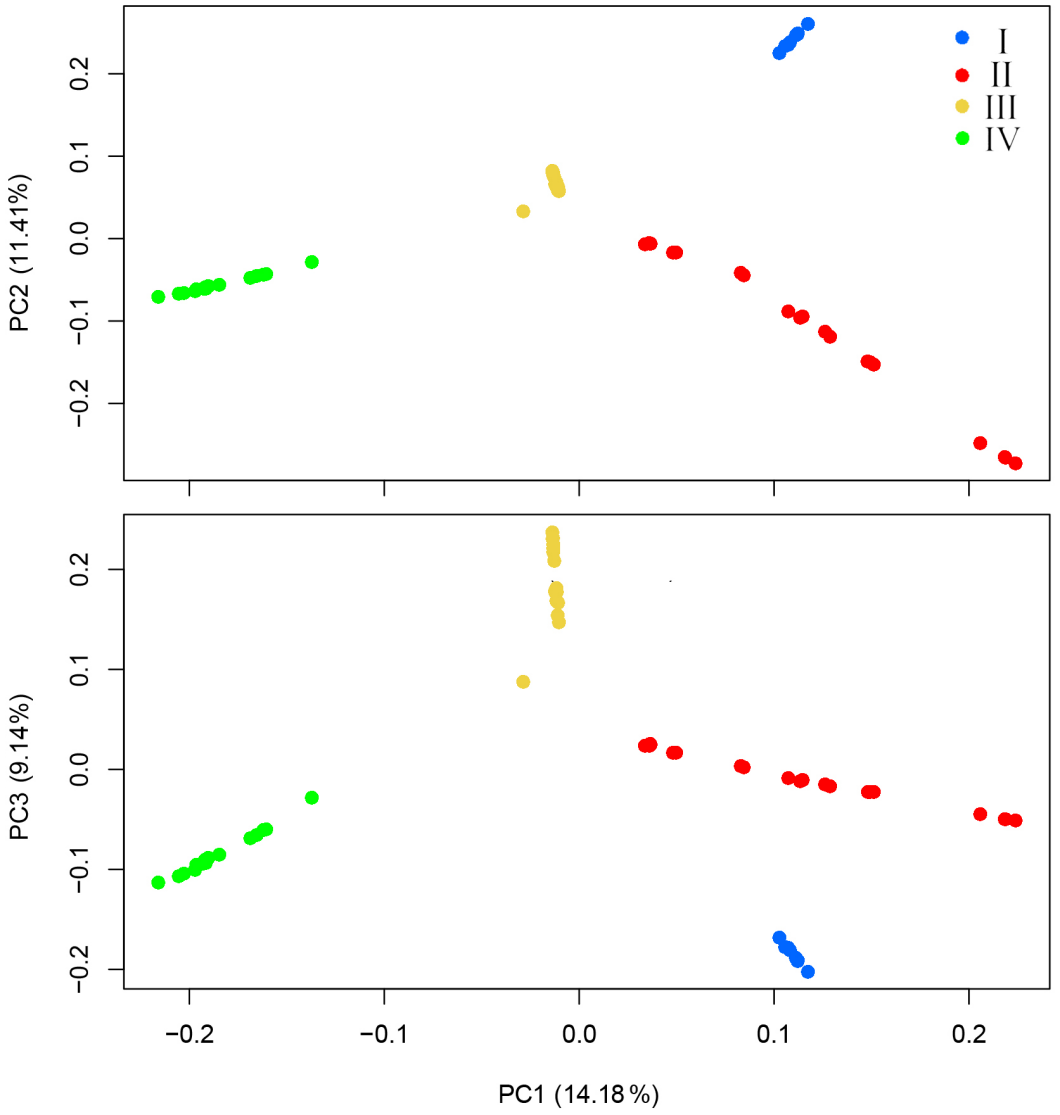
PCA showing population genetic structure and clustering among *Trimerodytes percarinatus* individuals based on SNP data. All genetic lineages are color‐coded as Figure 1.

**FIGURE 5:**
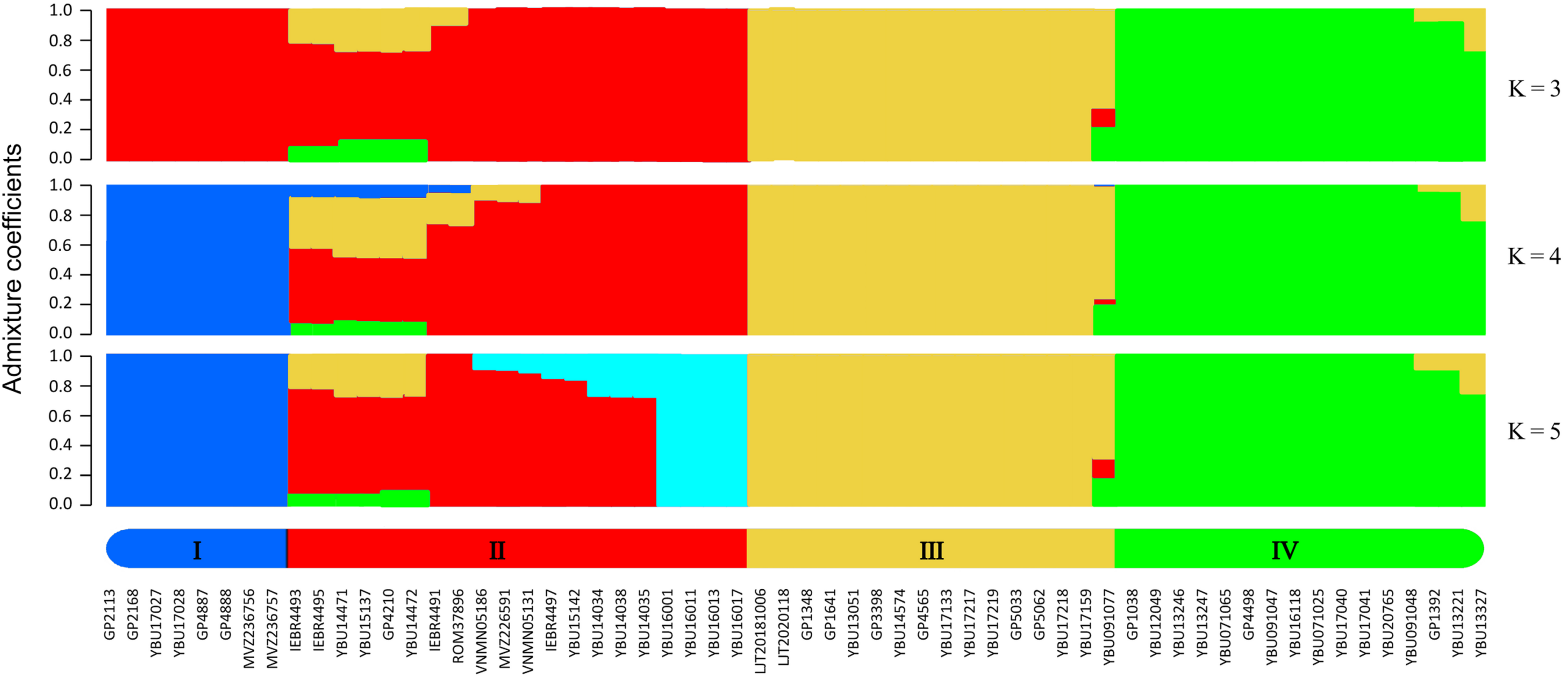
Population structure and admixture proportions of *Trimerodytes percarinatus* based on SNP data. All genetic lineages are color‐coded as Figure 1.

Results of the PCA and admixture analysis based on SNP data supported four distinct lineages within *T. percarinatus* showing strong geographic structure. In the PCA, the first, second, and third principal components (variances explained: 14.18%, 11.41% and 9.14%, respectively) separated this species into four different clusters (Figure 4). Admixture analyses found that K‐values ranging from 3 to 5 fit the data (Figure S1). When *K* = 4, the structure corresponded to the topology of the concatenated SNP gene tree (I–IV; Figures 3 and 5). The admixture analyses also demonstrated high levels of admixed ancestries for several sampled individuals.

### 3.3 Gene flow analyses

**FIGURE 6:**
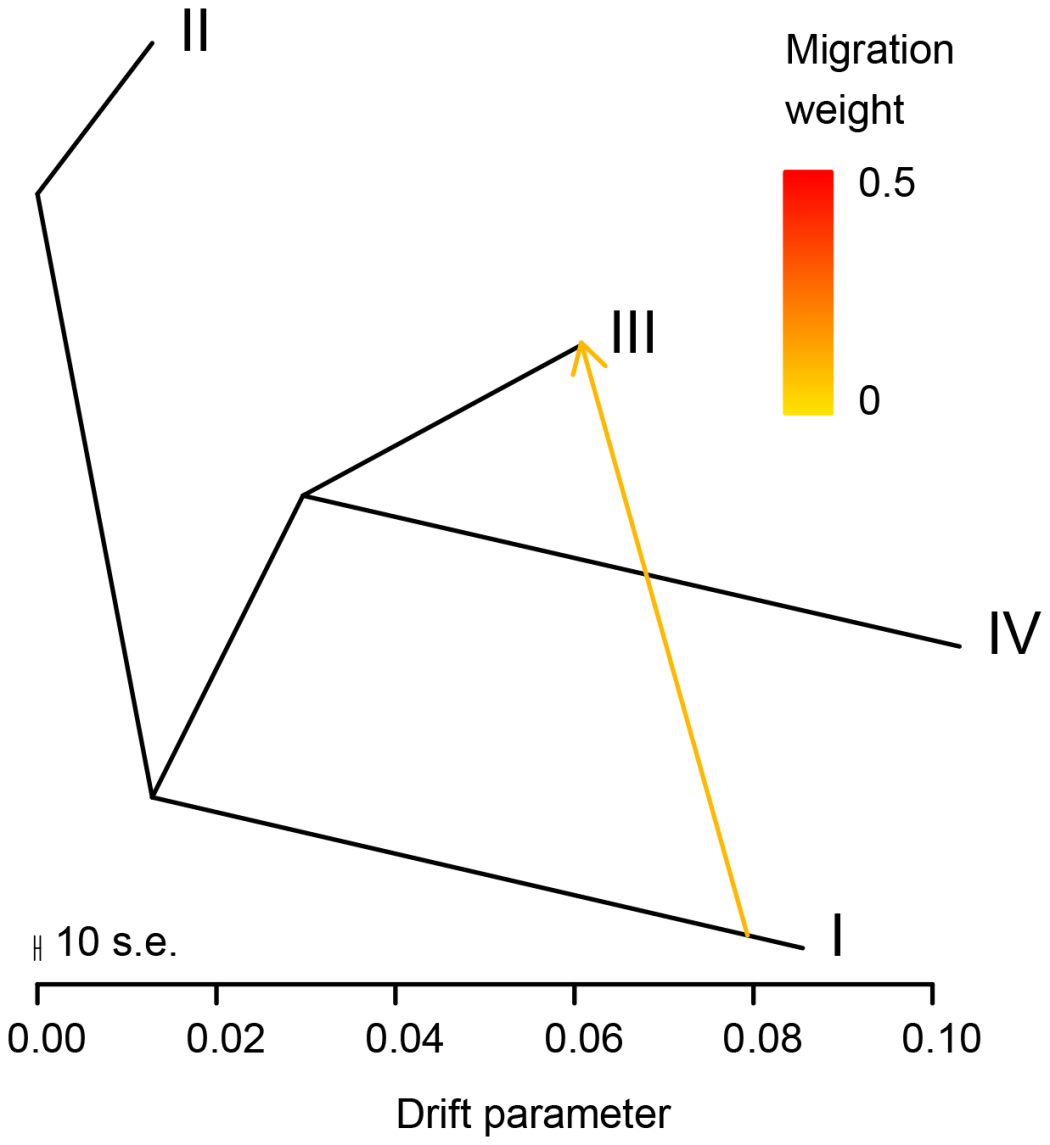
Treemix admixture graph with one migration event (m) from SNP data. The migration weight and the directionality of gene flow are indicated by the colored arrows. The graded bar on the right approximates color to values of migration weight as the fraction of ancestry derived from the migration source, so that the warmer color (dark red) indicates an ancestry of 50%. The scale below measures the genetic drift from the ancestral to the extant populations.

The results from the D and f4 statistics consistently revealed that all tests significantly deviated from neutrality (|Z‐score| > 3), suggesting the presence of gene flow between lineages I and III, as well as lineages II and IV (Table S5). In addition, comparing the likelihood support of the 5 different TreeMix runs suggested that there was only 1 instance of migration in the population tree model, which occurred between lineages I and III (Figure 6).

### 3.4 Divergence dating and ancestral area estimation

**FIGURE 7:**
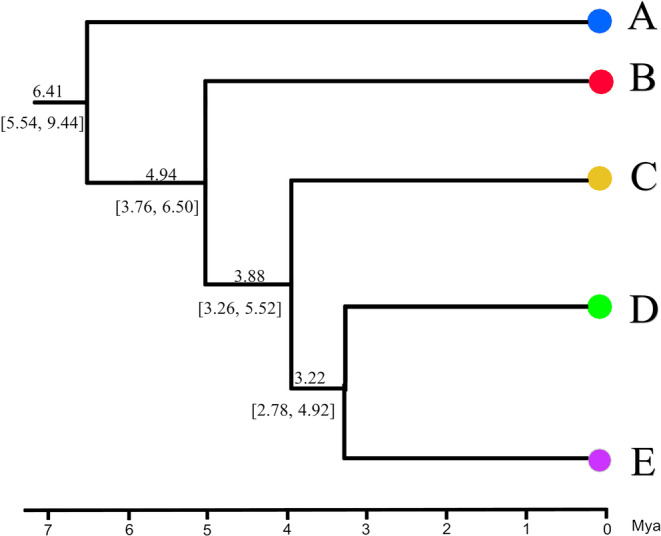
Divergence date estimation of *Trimerodytes percarinatus* using BEAST v2.6.6 with mtDNA loci. All genetic lineages are color‐coded as Figure 1.

Divergence dating suggested that *T. percarinatus* diverged from its sister taxon approximately 12.68 Mya [95% highest posterior density (HPD): 10.36–15.96 Mya] during the Mid‐late Miocene. Intraspecific divergence began around 6.41 Mya (95% HPD: 5.54–9.44 Mya) and most lineages were formed around 3.22–4.94 Mya (Figure 7). Ancestral area reconstruction indicated that *T. percarinatus* originated from a region encompassing much of southwestern China and Vietnam (SCV) (98% for S‐DIVA and 94% for DEC. Figure S2).

### 3.5 Historical demography

**FIGURE 8:**
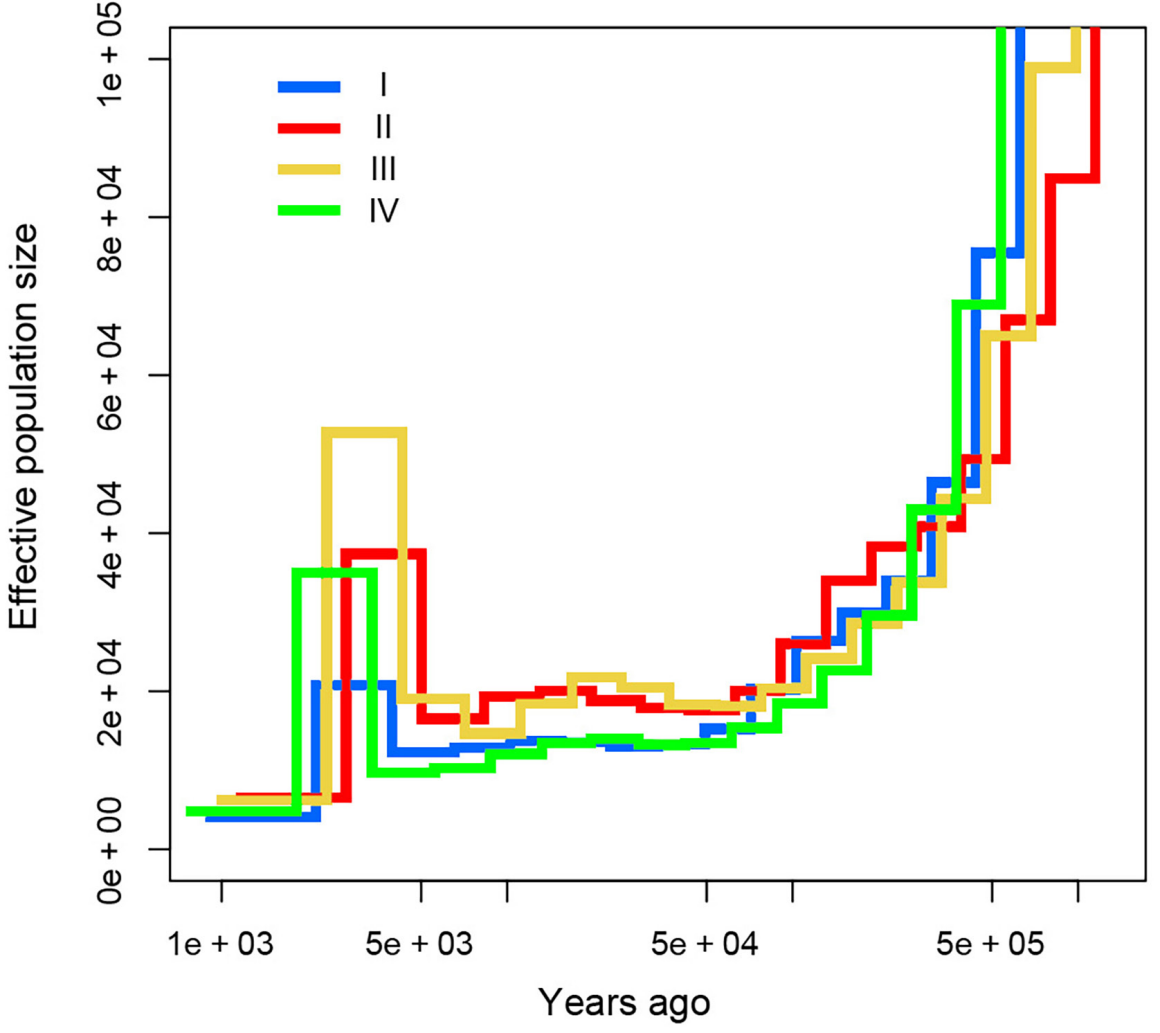
Demographic history of *Trimerodytes percarinatus* from SNP data. Time (in years) is shown along the x axis and effective population size is shown along the y axis. The generation time (g) is set to 3 years, and the generation mutation rate (μ) is 4.71 × 10^−9^ per site per generation. All genetic lineages are color‐coded as Figure 1.

MSMC2 analyses revealed continuous declines of effective population size (*Ne*) from 0.5 to 0.05 Mya for each analyzed lineage of *T. percarinatus* (Figure 8). Subsequently, *Ne* of the four lineages remained relatively stable from about 0.05 to 0.005 Mya. Then, all four lineages experienced a drastic rise in effective population size around 0.006 to 0.002 Mya, followed by a second sharp decline (Figure 8).

### 3.6 Ecological niche modeling

**FIGURE 9:**
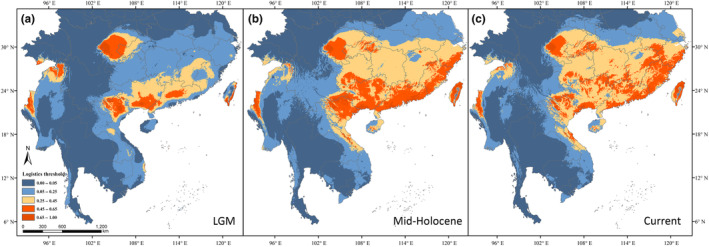
Inferred present and historical suitable habitats based on the ENMs of *Trimerodytes percarinatus* localities. Suitable habitats for *Trimerodytes percarinatus* during Last Glacial Maximum (a), Mid‐Holocene (b), and present (c) estimated using ENM. Warmer colors indicate regions with a higher logistic threshold probability of suitable habitat, while dark colors represent areas with lower probability of *Trimerodytes percarinatus* presence.

The AUC value approached 1 (≥0.98), indicating a good performance of the predictive models. The ENM results revealed that during the LGM, the Sichuan Basin, southern Guangdong and Guangxi, the south and east of Taiwan in China, northern Vietnam and northwestern Myanmar contained suitable environments and may have served as potential refugia (Figure 9A). During the Mid‐Holocene, suitable habitats extended southward to southern Vietnam and northward to the Yangtze River region in China (Figure 9B). The overall predicted distribution pattern was largely consistent with the current distribution of *T. percarinatus* (Figure 9C).

## 4 DISCUSSION

### 4.1 Phylogeographic patterns across East Asia

Snakes are generally susceptible to climate fluctuation and associated habitat changes over time. Although snake phylogeographic studies in China lag far behind those in North American and Europe, a few studies have revealed phylogeographic patterns across China (Guo et al., 2011, 2016, 2019; Huang et al., 2007; Zhu et al., 2016). However, these studies are mainly based on venomous snakes, particularly pitvipers. Our present study is the first work using a nonvenomous snake species with range‐wide sampling and genome‐based data. The results presented here demonstrate conclusively that *T. percarinatus* is composed of multiple ancient geographic lineages distributed across China and Vietnam.

Results from ancestral area estimation indicate that *T. percarinatus* originated in an area located in southwestern China and Vietnam. Previous studies based on snakes at different taxonomic levels consistently suggested that Qinghai‐Xizang Plateau (QXP) and Hengduan Mountains Region were the center of origin (Guo et al., 2011, 2016, 2020; Zhu et al., 2016). Our results for *T. percarinatus* are consistent with both this hypothesis and “out of Qinghai‐Xizang Plateau” and “out of Hengduan Mountains” (Guo et al., 2019; Päckert et al., 2020). The estimated origin date of this species is 12.68 Mya in the mid‐late Miocene, which is much older than some codistributed pitvipers (Guo et al., 2011, 2016), but similar to other colubrids (Guicking et al., 2006). Importantly, this date falls within the estimated time frame of the QXP uplift during the Miocene (25–10 Mya; Sun, 1997). The uplift of the QXP resulted in environmental transitions and historical climate change, and therefore may have facilitated *T. percarinatus* population divergence as seen in other taxa (Che et al., 2010; Fu et al., 2005; Guo et al., 2020; Zhu et al., 2022).

In East Asian snakes, phylogeographic studies have typically revealed the presence of both longitudinal and latitudinal diversification (e.g., Guo et al., 2011, 2020; Huang et al., 2007; Lin et al., 2014). Guo et al. (2020) suggested that longitudinal diversification occurred more commonly than latitudinal divergence across this region. Phylogenetic analyses based on the SNP dataset show that both diversification patterns were detected within *T. percarinatu*s. For example, latitudinal diversification had occurred between lineages I + II and III + IV (Figure 3). The Yunnan‐Guizhou Plateau, which is located between I + II and III + IV, may be responsible for this diversification. Lee (1939) and Jiang and Wu (1993) suggested that the Asian continent was composed of three geomorphologic steps, gradually decreasing in elevation from west to east. The longitudinal diversification pattern observed here between lineages III and IV is consistent with the boundary between the second and third geomorphologic steps. Similar patterns have also been uncovered in three Asian pitvipers (e.g., *Deinagkistrodon acutus*: Huang et al., 2007; *Protobothrops mucrosquamatus*: Guo et al., 2019; *Viridovipera stejnegeri*: Guo et al., 2016). These similar results suggest that differences in habitat between the second and the third geomorphologic steps may lead to population divergence within codistributed species. Of course, more phylogeographic studies on Chinese snakes with similar geographic distributions will provide additional insights into the drivers of lineage formation.

The geographic isolation of Hainan Island promoted lineage divergence between the local *T. percarinatus* population and those dispersed across mainland Southeast Asia. Hainan Island became geographically isolated from the Asian continent approximately 2.5 Mya (Shi et al., 2006; Zhao et al., 2007; Zhu, 2016). However, the estimated population divergence date indicates that the Hainan lineage of *T. percarinatus* originated ~6 Mya, suggesting that diversification predates the isolation of this island. Similar cases have been reported in several species of Asian pitvipers (Guo et al., 2016, 2019). Both SNP‐based phylogenetic analysis and admixture analysis indicate that the Hainan individuals are most closely related to the individuals from southern Guangxi, China and Vietnam, suggesting that the Hainan individuals may have been colonized from these regions, consistent with previous studies on other animal and plant groups (Chen et al., 2015; Lin et al., 2010; Zhu, 2016). Based on their work on snakes and previous studies on other organisms inhabiting in Hainan Island, Guo et al. (2016) proposed that there were two dispersal events contributing to the Hainan fauna and flora (Two Dispersals Hypothesis): one is from South China via the Qiongzhou Strait and the other from Vietnam via the Gulf of Tonkin. The case of Hainan individuals colonizing from Vietnam via the Gulf of Tonkin further confirms the hypotheses on Hainan Island fauna proposed by Guo et al. (2016).

### 4.2 Historical demography of *T. percarinatus*

Historical demography can be affected by various factors, including biological, such as life history, competitors, predation, parasitism, disease, human activities, and abiotic, such as geological processes, and glacial and interglacial periods. In Europe and North America, many organisms experienced changes in population size in response to the LGM (Guiher & Burbrink, 2008; Hewitt, 2004; Hull & Girman, 2005; Schield et al., 2018); however, this trend was not observed in numerous taxa distributed across East Asia (Gao et al., 2012; Guo et al., 2016, 2019; Huang et al., 2013; Zhou et al., 2013). Here, analyses of demographic history indicated that the effective population size experienced great changes in recent date. In the Middle Pleistocene (about 0.5–0.05 Mya), the formation of the Qinghai‐Xizang Plateau resulted in inland arid zone in Asia (Zhang, 2011). This unsuitable habitat has led to population declines in *T. percarinatus* lineages. In the subsequent LGM of the late Pleistocene (about 0.05Mya‐0.01Mya), the population sizes remained stable, indicating the effect of the LGM on the effective population size of *T. percarinatus* was not significant, which may be due to the refugia provided in the south region of the Yangtze River. ENMs confirmed the existence of several refugias in southwest China, southern China, and Southeast Asia (Figure 9A). Several population demographic studies across East Asia uncovered that some organisms with similar distribution have not been influenced by past climate change events associated with glacial cycles (e.g., *Nanorana yunnanensis*: Zhang et al., 2010; *Quasipaa boulengeri*: Yan et al., 2013; *Sarcocheilichthys sinensis*: Ding et al., 2020; *Odorrana graminea*: Chen et al., 2020). In the Middle Holocene (0.007–0.0025 Mya), a warm climate led to population expansion (Figure 8. Marcott et al., 2013; Wu et al., 2023).

### 4.3 Intraspecific divergence within *T. percarinatus* and taxonomic implications

A high degree of intraspecific genetic diversity can be indicative of a species' ability to respond and adapt to changing habitats and environmental stressors. In this study, genomic sequence data demonstrated high genetic diversity, and the presence of several geographically structured lineages within *T. percarinatus*. The high genetic diversity and pronounced population structure in *T. percarinatus* may be attributed to several different factors. As a semiaquatic species, which prefers small rivers, streams, and ponds, the dispersal ability of *T. percarinatus* is contingent upon drainage systems and river networks. Therefore, the lack of these aquatic features in terrestrial ecosystems may act as a physical barrier to gene flow between populations, thereby promoting genetic differentiation (e.g., Yan et al., 2013; Zhou et al., 2013). Population structure analyses (Figure 5) indicated that there was no significant gene flow among lineages. Second, *T. percarinatus* typically inhabits regions at elevations below 1000 m (rarely exceeding 2000 m), and therefore mountain ranges may also prevent dispersal.

The population that occurs on Taiwan, long recognized as the subspecies *T. p. suriki* (Maki), was represented in this study by a single sample (GP 5122). Despite the lack of SNP data generated for this sample, the mtDNA‐based phylogeny indicated that it was nested within the clade of all samples collected from the Chinese mainland, resulting in the nonmonophyly of the nominal subspecies. Furthermore, the Taiwanese *T. p. suriki* ventral scale counts (range: 142–153) are not significantly divergent from its closest relatives *T. p. percarinatus* (range: 131–160) in China (Zhao et al., 1998). Thus, based on this evidence, we question the validity of the subspecies *T. p. suriki*, and suggest that further investigation is needed.

Both mtDNA‐based and SNP‐based analyses consistently uncovered the presence of deep divergent lineages within *T. percarinatus* (e.g., lineages I + II and III + IV). Whether these deep lineages represent distinct species is unknown and future studies with more sampling, additional genomic data, and more detailed morphological investigations will be necessary to address this question.

## AUTHOR CONTRIBUTIONS

**Bing Lyu:** Conceptualization (lead); data curation (lead); formal analysis (lead); investigation (lead); methodology (lead); validation (equal); visualization (equal); writing – original draft (lead); writing – review and editing (equal). **Qin Liu:** Data curation (supporting); formal analysis (supporting); resources (equal). **Yayong Wu:** Data curation (supporting); investigation (supporting); writing – review and editing (supporting). **Truong Q. Nguyen:** Data curation (supporting); investigation (supporting); writing – review and editing (supporting). **Jing Che:** Data curation (supporting); formal analysis (supporting); investigation (supporting); writing – review and editing (supporting). **Sang N. Nguyen:** Data curation (supporting); investigation (supporting); writing – review and editing (supporting). **Edward A. Myers:** Formal analysis (supporting); writing – review and editing (supporting). **Frank T. Burbrink:** Formal analysis (supporting); writing – review and editing (supporting). **Peng Guo:** Conceptualization (lead); data curation (equal); funding acquisition (lead); project administration (lead); resources (lead); supervision (lead); validation (equal); writing – original draft (supporting); writing – review and editing (equal). **Jichao Wang:** Conceptualization (equal); data curation (equal); formal analysis (supporting); investigation (supporting); methodology (supporting); supervision (equal); validation (equal); visualization (equal); writing – review and editing (equal).

## FUNDING INFORMATION

This project was supported by grants from the Second Tibetan Plateau Scientific Expedition and Research (STEP) program (2019QZKK05010105), Southeast Asia Biodiversity Research Institute, CAS, the Digitalization, Development and Application of Biotic Resource (202002AA100007), China's Biodiversity Observation Network (Sino‐BON), Animal Branch of the Germplasm Bank of Wild Species, CAS (Large Research Infrastructure Funding), Sciences and Technology Department of Sichuan Province (2020YFSY0033). Research of T.Q. Nguyen was supported by the National Foundation for Science and Technology Development (NAFOSTED) (106.05–2021.19).

## CONFLICT OF INTEREST STATEMENT

The authors declare no competing interests.

## Supporting information


Figure S1.

Figure S2.

Table S1.

Table S2.

Table S3.

Table S4.

Table S5.

## Data Availability

The mtDNA sequences newly generated are deposited in GenBank (accession nos.: OQ575713–OQ576010). All raw sequencing reads are available at the National Center for Biotechnology Information Sequence Read Archive under accession number PRJNA1078921.
