# Supplementary figures and images for: Genomic analysis reveals deep population divergence in the water snake Trimerodytes percarinatus (Serpentes, Natricidae)
Source: Ecol Evol. 2024 Apr 15;14(4):e11278. doi: 10.1002/ece3.11278 (PMC11019134; doi:10.1002/ece3.11278)

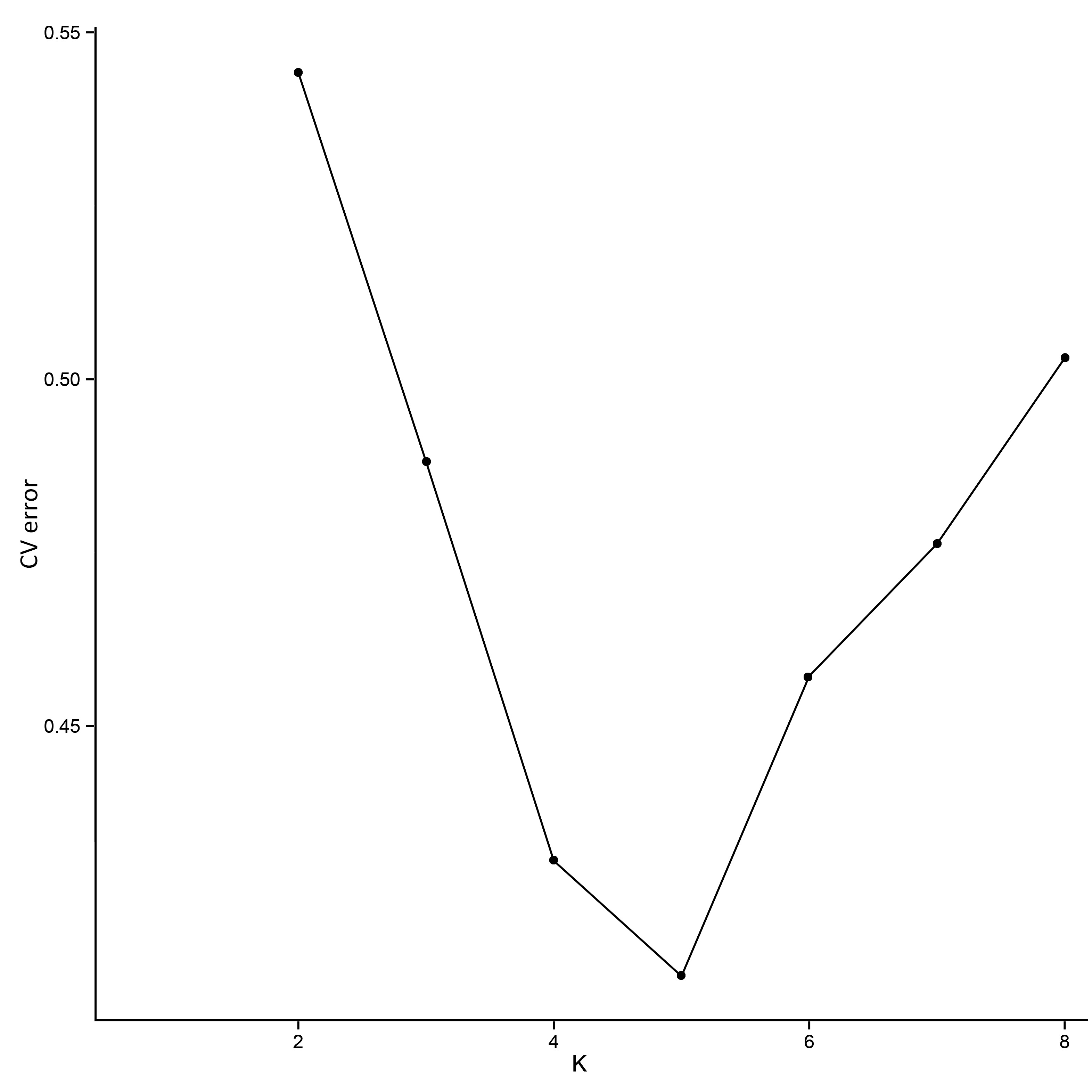

Supplement: Supplementary file 1 — Figure S1. [file ECE3-14-e11278-s004.jpg]

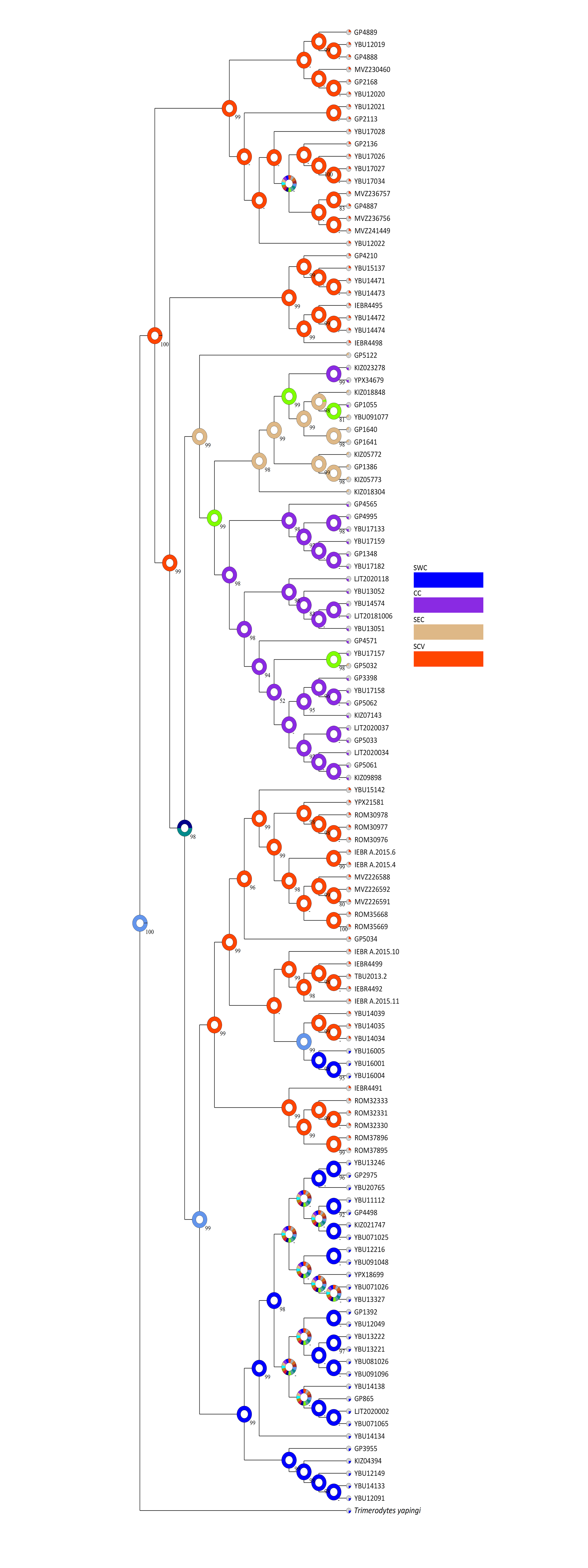

Supplement: Supplementary file 2 — Figure S2. [file ECE3-14-e11278-s002.tif]
