# Supplementary material for: Genomic analysis reveals deep population divergence in the water snake Trimerodytes percarinatus (Serpentes, Natricidae)
Source: Ecol Evol. 2024 Apr 15;14(4):e11278. doi: 10.1002/ece3.11278 (PMC11019134; doi:10.1002/ece3.11278)
Supplement: Supplementary file 3 — Table S1. [file ECE3-14-e11278-s005.docx]

Table S1 Detailed information of samples used in molecular phylogeny.

| **ID** | **Species name** | **Voucher number** | **Locality** | **Accession number (cyt *b*/ND2)** | | **Sequence source** |
| --- | --- | --- | --- | --- | --- | --- |
|  | *Trimerodytes percarinatus* | GP2136 | Diaoluo Mountain, Hainan, China | OQ575713 | OQ575860 | In this study |
|  | *Trimerodytes percarinatus* | YBU12019 | Diaoluo Mountain, Hainan, China | OQ575714 | OQ575861 | In this study |
|  | *Trimerodytes percarinatus* | YBU12020 | Diaoluo Mountain, Hainan, China | OQ575715 | OQ575862 | In this study |
|  | *Trimerodytes percarinatus* | YBU12021 | Diaoluo Mountain, Hainan, China | OQ575716 | OQ575863 | In this study |
|  | *Trimerodytes percarinatus* | YBU12022 | Diaoluo Mountain, Hainan, China | OQ575717 | OQ575864 | In this study |
|  | *Trimerodytes percarinatus* | GP2168* | Diaoluo Mountain, Hainan, China | OQ575718 | OQ575865 | In this study |
|  | *Trimerodytes percarinatus* | YBU17026 | Jianfengling Mountain, Hainan, China | OQ575719 | OQ575866 | In this study |
|  | *Trimerodytes percarinatus* | YBU17027* | Jianfengling Mountain, Hainan, China | OQ575720 | OQ575867 | In this study |
|  | *Trimerodytes percarinatus* | YBU17028* | Jianfengling Mountain, Hainan, China | OQ575721 | OQ575868 | In this study |
|  | *Trimerodytes percarinatus* | YBU17034 | Jianfengling Mountain, Hainan, China | OQ575722 | OQ575869 | In this study |
|  | *Trimerodytes percarinatus* | GP4887* | Limu Mountain, Hainan, China | OQ575723 | OQ575870 | In this study |
|  | *Trimerodytes percarinatus* | GP4888* | Limu Mountain, Hainan, China | OQ575724 | OQ575871 | In this study |
|  | *Trimerodytes percarinatus* | GP4889 | Limu Mountain, Hainan, China | OQ575725 | OQ575872 | In this study |
|  | *Trimerodytes percarinatus* | MVZ230460 | Qiongzhong, Hainan, China | OQ575726 | OQ575873 | In this study |
|  | *Trimerodytes percarinatus* | MVZ236756* | Bawangling Mountain, Hainan, China | OQ575727 | OQ575874 | In this study |
|  | *Trimerodytes percarinatus* | MVZ236757* | Bawangling Mountain, Hainan, China | OQ575728 | OQ575875 | In this study |
|  | *Trimerodytes percarinatus* | MVZ241449 | Qiongzhong, Hainan, China | OQ575729 | OQ575876 | In this study |
|  | *Trimerodytes percarinatus* | YBU14471* | Fangchenggang , Guangxi, China | OQ575730 | OQ575877 | In this study |
|  | *Trimerodytes percarinatus* | YBU14472* | Fangchenggang , Guangxi, China | OQ575731 | OQ575878 | In this study |
|  | *Trimerodytes percarinatus* | YBU14473 | Fangchenggang , Guangxi, China | OQ575732 | OQ575879 | In this study |
|  | *Trimerodytes percarinatus* | GP4210* | Wangle, Guangxi, China | OQ575733 | OQ575880 | In this study |
|  | *Trimerodytes percarinatus* | YBU15137* | Wangle, Guangxi, China | OQ575734 | OQ575881 | In this study |
|  | *Trimerodytes percarinatus* | IEBR4495* | Quang Ninh, Vietnam | OQ575735 | OQ575882 | In this study |
|  | *Trimerodytes percarinatus* | GP1798 | - | OQ575736 | OQ575883 | In this study |
|  | *Trimerodytes percarinatus* | YBU13052 | Wuyishan, Fujian, China | OQ575737 | OQ575884 | In this study |
|  | *Trimerodytes percarinatus* | GP3398* | Huangshan, Anhui, China | OQ575738 | OQ575885 | In this study |
|  | *Trimerodytes percarinatus* | YBU14574* | Shangrao, Jiangxi, China | OQ575739 | OQ575886 | In this study |
|  | *Trimerodytes percarinatus* | GP4565* | Xianju, Zhejiang, China | OQ575740 | OQ575887 | In this study |
|  | *Trimerodytes percarinatus* | GP4571 | Suichang, Zhejiang, China | OQ575741 | OQ575888 | In this study |
|  | *Trimerodytes percarinatus* | YBU17133* | Hangzhout, Zhejiang, China | OQ575742 | OQ575889 | In this study |
|  | *Trimerodytes percarinatus* | YBU17157 | Dongyang, Zhejiang, China | OQ575743 | OQ575890 | In this study |
|  | *Trimerodytes percarinatus* | YBU17158 | Wenzhou, Zhejiang, China | OQ575744 | OQ575891 | In this study |
|  | *Trimerodytes percarinatus* | YBU17159* | Hangzhou, Zhejiang, China | OQ575745 | OQ575892 | In this study |
|  | *Trimerodytes percarinatus* | YBU17182 | Dongyang, Zhejiang, China | OQ575746 | OQ575893 | In this study |
|  | *Trimerodytes percarinatus* | YBU17217* | Wenzhou, Zhejiang, China | OQ575747 | OQ575894 | In this study |
|  | *Trimerodytes percarinatus* | YBU17218* | Wenzhou, Zhejiang, China | OQ575748 | OQ575895 | In this study |
|  | *Trimerodytes percarinatus* | YBU17219* | Wenzhou, Zhejiang, China | OQ575749 | OQ575896 | In this study |
|  | *Trimerodytes percarinatus* | GP4995 | Dongyang, Zhejiang, China | OQ575750 | OQ575897 | In this study |
|  | *Trimerodytes percarinatus* | GP5032 | Ningde, Fujian, China | OQ575751 | OQ575898 | In this study |
|  | *Trimerodytes percarinatus* | GP5033* | Quanzhou, Fujian, China | OQ575752 | OQ575899 | In this study |
|  | *Trimerodytes percarinatus* | GP5061 | Huangshan, Anhui, China | - | OQ575900 | In this study |
|  | *Trimerodytes percarinatus* | GP5062* | Huangshan, Anhui, China | OQ575753 | OQ575901 | In this study |
|  | *Trimerodytes percarinatus* | KIZ 05772 | Hong Kong, China | OQ575754 | OQ575902 | In this study |
|  | *Trimerodytes percarinatus* | KIZ 05773 | Hong Kong, China | OQ575755 | OQ575903 | In this study |
|  | *Trimerodytes percarinatus* | KIZ 09898 | Quanzhou, Fujian, China | OQ575756 | OQ575904 | In this study |
|  | *Trimerodytes percarinatus* | KIZ 07143 | Yichun, Jiangxi, China | OQ575757 | OQ575905 | In this study |
|  | *Trimerodytes percarinatus* | KIZ 023278 | Xinyang, Henan, China | OQ575758 | OQ575906 | In this study |
|  | *Trimerodytes percarinatus* | KIZ YPX34679 | Xinyang, Henan, China | OQ575759 | OQ575907 | In this study |
|  | *Trimerodytes percarinatus* | KIZ 018848 | Qingyuan, Guangdong, China | OQ575760 | OQ575908 | In this study |
|  | *Trimerodytes percarinatus* | KIZ 018304 | Yunfu, Guangdong, China | OQ575761 | OQ575909 | In this study |
|  | *Trimerodytes percarinatus* | LJT2020034 | Sanming, Fujian, China | OQ575762 | - | In this study |
|  | *Trimerodytes percarinatus* | LJT2020037 | Sanming, Fujian, China | - | OQ575910 | In this study |
|  | *Trimerodytes percarinatus* | LJT20181006* | Wuyishan, Fujian, China | OQ575763 | OQ575911 | In this study |
|  | *Trimerodytes percarinatus* | LJT2020118* | Ji'an, Jiangxi, China | OQ575764 | OQ575912 | In this study |
|  | *Trimerodytes percarinatus* | GP5122 | Taiwan, China | OQ575765 | OQ575913 | In this study |
|  | *Trimerodytes percarinatus* | YBU20765* | Pingshan, Sichuan, China | OQ575766 | OQ575914 | In this study |
|  | *Trimerodytes percarinatus* | YBU11112 | Hejiang, Sichuan, China | OQ575767 | OQ575915 | In this study |
|  | *Trimerodytes percarinatus* | YBU12049* | Yibin, Sichuan, China | OQ575768 | OQ575916 | In this study |
|  | *Trimerodytes percarinatus* | YBU12091 | Leishan, Guizhou, China | OQ575769 | OQ575917 | In this study |
|  | *Trimerodytes percarinatus* | YBU12149 | Rongjiang, Guizhou, China | OQ575770 | OQ575918 | In this study |
|  | *Trimerodytes percarinatus* | YBU12216 | Rongjiang, Guizhou, China | OQ575771 | OQ575919 | In this study |
|  | *Trimerodytes percarinatus* | YBU13221* | Tongren, Guizhou, China | OQ575772 | OQ575920 | In this study |
|  | *Trimerodytes percarinatus* | YBU13222 | Tongren, Guizhou, China | OQ575773 | OQ575921 | In this study |
|  | *Trimerodytes percarinatus* | YBU13247* | Yinjiang, Guizhou, China | OQ575774 | OQ575922 | In this study |
|  | *Trimerodytes percarinatus* | GP2975 | Yinjiang, Guizhou, China | OQ575775 | OQ575923 | In this study |
|  | *Trimerodytes percarinatus* | YBU13327* | Wufeng, Hubei, China | OQ575776 | OQ575924 | In this study |
|  | *Trimerodytes percarinatus* | YBU14133 | Liping, Guizhou, China | OQ575777 | OQ575925 | In this study |
|  | *Trimerodytes percarinatus* | YBU14134 | Leishan, Guizhou, China | OQ575778 | OQ575926 | In this study |
|  | *Trimerodytes percarinatus* | YBU14138 | Yibin, Sichuan, China | OQ575779 | OQ575927 | In this study |
|  | *Trimerodytes percarinatus* | GP3955 | Guilin, Guangxi, China | OQ575780 | OQ575928 | In this study |
|  | *Trimerodytes percarinatus* | GP4498* | Hejiang, Sichuan, China | OQ575781 | OQ575929 | In this study |
|  | *Trimerodytes percarinatus* | YBU16118* | Junlian, Sichuan, China | OQ575782 | OQ575930 | In this study |
|  | *Trimerodytes percarinatus* | YBU17040* | Junlian, Sichuan, China | OQ575783 | OQ575931 | In this study |
|  | *Trimerodytes percarinatus* | YBU17041* | Junlian, Sichuan, China | OQ575784 | OQ575932 | In this study |
|  | *Trimerodytes percarinatus* | KIZ YPX18699 | Chongqing, China | OQ575785 | OQ575933 | In this study |
|  | *Trimerodytes percarinatus* | KIZ021747 | Rongshui, Guangxi, China | OQ575786 | OQ575934 | In this study |
|  | *Trimerodytes percarinatus* | KIZ04394 | Huaihua, Hunan, China | OQ575787 | OQ575935 | In this study |
|  | *Trimerodytes percarinatus* | LJT2020002 | Dujiangyan, Sichuan, China | OQ575788 | OQ575936 | In this study |
|  | *Trimerodytes percarinatus* | YBU14035* | Jinping, Yunnan, China | OQ575789 | OQ575937 | In this study |
|  | *Trimerodytes percarinatus* | YBU14036 | Jinping, Yunnan, China | OQ575790 | OQ575938 | In this study |
|  | *Trimerodytes percarinatus* | YBU14037 | Jinping, Yunnan, China | OQ575791 | OQ575939 | In this study |
|  | *Trimerodytes percarinatus* | YBU14038* | Jinping, Yunnan, China | OQ575792 | OQ575940 | In this study |
|  | *Trimerodytes percarinatus* | YBU14039 | Jinping, Yunnan, China | OQ575793 | OQ575941 | In this study |
|  | *Trimerodytes percarinatus* | YBU15142* | Maguan, Yunnan, China | OQ575794 | OQ575942 | In this study |
|  | *Trimerodytes percarinatus* | YBU16001* | Shuangbai, Yunnan, China | OQ575795 | OQ575943 | In this study |
|  | *Trimerodytes percarinatus* | YBU16004 | Shuangbai, Yunnan, China | OQ575796 | OQ575944 | In this study |
|  | *Trimerodytes percarinatus* | YBU16005 | Shuangbai, Yunnan, China | OQ575797 | OQ575945 | In this study |
|  | *Trimerodytes percarinatus* | YBU16006 | Shuangbai, Yunnan, China | OQ575798 | OQ575946 | In this study |
|  | *Trimerodytes percarinatus* | YBU16007 | Shuangbai, Yunnan, China | OQ575799 | OQ575947 | In this study |
|  | *Trimerodytes percarinatus* | YBU16008 | Shuangbai, Yunnan, China | OQ575800 | OQ575948 | In this study |
|  | *Trimerodytes percarinatus* | YBU16010 | Shuangbai, Yunnan, China | OQ575801 | OQ575949 | In this study |
|  | *Trimerodytes percarinatus* | YBU16011* | Shuangbai, Yunnan, China | OQ575802 | OQ575950 | In this study |
|  | *Trimerodytes percarinatus* | YBU16012 | Shuangbai, Yunnan, China | OQ575803 | OQ575951 | In this study |
|  | *Trimerodytes percarinatus* | YBU16013* | Shuangbai, Yunnan, China | OQ575804 | OQ575952 | In this study |
|  | *Trimerodytes percarinatus* | YBU16014 | Shuangbai, Yunnan, China | OQ575805 | OQ575953 | In this study |
|  | *Trimerodytes percarinatus* | YBU16015 | Shuangbai, Yunnan, China | OQ575806 | OQ575954 | In this study |
|  | *Trimerodytes percarinatus* | YBU16016 | Shuangbai, Yunnan, China | OQ575807 | OQ575955 | In this study |
|  | *Trimerodytes percarinatus* | YBU16017* | Shuangbai, Yunnan, China | OQ575808 | OQ575956 | In this study |
|  | *Trimerodytes percarinatus* | YBU16018 | Shuangbai, Yunnan, China | OQ575809 | OQ575957 | In this study |
|  | *Trimerodytes percarinatus* | YBU16019 | Shuangbai, Yunnan, China | OQ575810 | OQ575958 | In this study |
|  | *Trimerodytes percarinatus* | YBU16020 | Shuangbai, Yunnan, China | OQ575811 | OQ575959 | In this study |
|  | *Trimerodytes percarinatus* | GP5034 | Maguan, Yunnan, China | OQ575812 | OQ575960 | In this study |
|  | *Trimerodytes percarinatus* | ROM30980 | Nghe An, Vietnam | OQ575813 | OQ575961 | In this study |
|  | *Trimerodytes percarinatus* | ROM30979 | Nghe An, Vietnam | OQ575814 | OQ575962 | In this study |
|  | *Trimerodytes percarinatus* | ROM30978 | Nghe An, Vietnam | OQ575815 | OQ575963 | In this study |
|  | *Trimerodytes percarinatus* | ROM30977 | Nghe An, Vietnam | OQ575816 | OQ575964 | In this study |
|  | *Trimerodytes percarinatus* | ROM30976 | Nghe An, Vietnam | OQ575817 | OQ575965 | In this study |
|  | *Trimerodytes percarinatus* | ROM31054 | Vinh Phuc, Vietnam | - | OQ575966 | In this study |
|  | *Trimerodytes percarinatus* | ROM35669 | Nguyen Binh, Cao Bang, Vietnam | OQ575818 | OQ575967 | In this study |
|  | *Trimerodytes percarinatus* | ROM35668 | Nguyen Binh, Cao Bang, Vietnam | OQ575819 | OQ575968 | In this study |
|  | *Trimerodytes percarinatus* | MVZ224254 | Vinh Phuc, Vietnam | OQ575820 | OQ575969 | In this study |
|  | *Trimerodytes percarinatus* | MVZ224256 | Vinh Phuc, Vietnam | OQ575821 | OQ575970 | In this study |
|  | *Trimerodytes percarinatus* | MVZ226588 | Vinh Phuc, Vietnam | OQ575822 | OQ575971 | In this study |
|  | *Trimerodytes percarinatus* | MVZ226589 | Vinh Phuc, Vietnam | OQ575823 | OQ575972 | In this study |
|  | *Trimerodytes percarinatus* | MVZ226590 | Vinh Phuc, Vietnam | OQ575824 | OQ575973 | In this study |
|  | *Trimerodytes percarinatus* | MVZ226591* | Vinh Phuc, Vietnam | OQ575825 | OQ575974 | In this study |
|  | *Trimerodytes percarinatus* | MVZ226592 | Vinh Phuc, Vietnam | OQ575826 | OQ575975 | In this study |
|  | *Trimerodytes percarinatus* | MVZ226593 | Vinh Phuc, Vietnam | OQ575827 | OQ575976 | In this study |
|  | *Trimerodytes percarinatus* | IEBRA.2015.10 | Dien Bien, Vietnam | OQ575828 | OQ575977 | In this study |
|  | *Trimerodytes percarinatus* | TBU2013.2 | Son La, Vietnam | OQ575829 | OQ575978 | In this study |
|  | *Trimerodytes percarinatus* | IEBR4499 | Son La, Vietnam | OQ575830 | OQ575979 | In this study |
|  | *Trimerodytes percarinatus* | TBU2014.8 | Son La, Vietnam | OQ575831 | OQ575980 | In this study |
|  | *Trimerodytes percarinatus* | TBU2014.7 | Son La, Vietnam | OQ575832 | OQ575981 | In this study |
|  | *Trimerodytes percarinatus* | IEBRA.2015.4 | Tsinghua, Vietnam | OQ575833 | OQ575982 | In this study |
|  | *Trimerodytes percarinatus* | IEBRA.2015.5 | Tsinghua, Vietnam | OQ575834 | OQ575983 | In this study |
|  | *Trimerodytes percarinatus* | IEBRA.2015.6 | Tsinghua, Vietnam | OQ575835 | OQ575984 | In this study |
|  | *Trimerodytes percarinatus* | IEBRA.2015.7 | Tsinghua, Vietnam | - | OQ575985 | In this study |
|  | *Trimerodytes percarinatus* | IEBR4492 | Bac Giang, Vietnam | OQ575836 | OQ575986 | In this study |
|  | *Trimerodytes percarinatus* | IEBR4496 | Son La, Vietnam | OQ575837 | OQ575987 | In this study |
|  | *Trimerodytes percarinatus* | IEBR4498 | Son La, Vietnam | OQ575838 | OQ575988 | In this study |
|  | *Trimerodytes percarinatus* | VNMN04912 | Nghe An, Vietnam | OQ575839 | OQ575989 | In this study |
|  | *Trimerodytes percarinatus* | VNMN05131* | Nghe An, Vietnam | OQ575840 | OQ575990 | In this study |
|  | *Trimerodytes percarinatus* | VNMN05186* | Nghe An, Vietnam | OQ575841 | OQ575991 | In this study |
|  | *Trimerodytes percarinatus* | VNMN05199 | Nghe An, Vietnam | OQ575842 | OQ575992 | In this study |
|  | *Trimerodytes percarinatus* | TBU2015.13 | Son La, Vietnam | OQ575843 | - | In this study |
|  | *Trimerodytes percarinatus* | IEBR4497* | Son La, Vietnam | OQ575844 | OQ575993 | In this study |
|  | *Trimerodytes percarinatus* | TBU2015.14 | Son La, Vietnam | - | OQ575994 | In this study |
|  | *Trimerodytes percarinatus* | KIZ YPX21581 | Quang Binh, Vietnam | OQ575845 | OQ575995 | In this study |
|  | *Trimerodytes percarinatus* | VNMN06215 | Vinh Phuc, Vietnam | OQ575846 | OQ575996 | In this study |
|  | *Trimerodytes percarinatus* | ROM32333 | Gia Lai, Vietnam | OQ575847 | OQ575997 | In this study |
|  | *Trimerodytes percarinatus* | ROM32332 | Gia Lai, Vietnam | OQ575848 | OQ575998 | In this study |
|  | *Trimerodytes percarinatus* | ROM32331 | Gia Lai, Vietnam | OQ575849 | OQ575999 | In this study |
|  | *Trimerodytes percarinatus* | ROM32330 | Gia Lai, Vietnam | OQ575850 | OQ576000 | In this study |
|  | *Trimerodytes percarinatus* | ROM30767 | Gia Lai, Vietnam | OQ575851 | OQ576001 | In this study |
|  | *Trimerodytes percarinatus* | ROM30992 | Gia Lai, Vietnam | OQ575852 | OQ576002 | In this study |
|  | *Trimerodytes percarinatus* | ROM37896* | Kon Tum, Vietnam | OQ575853 | OQ576003 | In this study |
|  | *Trimerodytes percarinatus* | ROM37895 | Kon Tum, Vietnam | OQ575854 | OQ576004 | In this study |
|  | *Trimerodytes percarinatus* | IEBRA.2015.8 | Gia Lai, Vietnam | OQ575855 | OQ576005 | In this study |
|  | *Trimerodytes percarinatus* | IEBR4490 | Gia Lai, Vietnam | - | OQ576006 | In this study |
|  | *Trimerodytes percarinatus* | IEBRA.2015.9 | Gia Lai, Vietnam | OQ575856 | OQ576007 | In this study |
|  | *Trimerodytes percarinatus* | IEBR4491* | Quang Binh, Vietnam | OQ575857 | OQ576008 | In this study |
|  | *Trimerodytes percarinatus* | VNMN2018154 | Gia Lai, Vietnam | OQ575858 | OQ576009 | In this study |
|  | *Trimerodytes yunnanensis* | CAS221544 | Myanmar | OQ575859 | OQ576010 | In this study |
|  | *Trimerodytes percarinatus* | GP2113* | Diaoluo Mountain, Hainan, China | MN582478 | MN582520 | Guo et al., 2020 |
|  | *Trimerodytes percarinatus* | YBU14474/GP3478 | Fangchenggang , Guangxi, China | MN582484 | MN582526 | Guo et al., 2020 |
|  | *Trimerodytes percarinatus* | IEBR4493* | Quang Ninh, Vietnam | MN582500 | MN582544 | Guo et al., 2020 |
|  | *Trimerodytes percarinatus* | YBU091077/GP996* | Tongle, Guangxi, China | MN582497 | MN582541 | Guo et al., 2020 |
|  | *Trimerodytes percarinatus* | GP1055 | Yizhang, Hunan, China | MN582469 | MN582511 | Guo et al., 2020 |
|  | *Trimerodytes percarinatus* | GP1348* | Tiantai, Zhejiang, China | MN582472 | MN582514 | Guo et al., 2020 |
|  | *Trimerodytes percarinatus* | GP1386 | Hong Kong, China | MN582473 | MN582515 | Guo et al., 2020 |
|  | *Trimerodytes percarinatus* | GP1640 | Conghua,Guangdong, China | MN582476 | MN582518 | Guo et al., 2020 |
|  | *Trimerodytes percarinatus* | GP1641* | Conghua,Guangdong, China | MN582477 | MN582519 | Guo et al., 2020 |
|  | *Trimerodytes percarinatus* | YBU13051/GP2749* | Wuyishan, Fujian, China | MN582479 | MN582521 | Guo et al., 2020 |
|  | *Trimerodytes percarinatus* | YBU071025/GP324* | Junlian, Sichuan, China | GQ281784 | JQ687455 | Guo et al., 2020 |
|  | *Trimerodytes percarinatus* | YBU071026/GP325 | Junlian, Sichuan, China | MN582481 | MN582523 | Guo et al., 2020 |
|  | *Trimerodytes percarinatus* | YBU071065/GP431* | Hejiang, Sichun, China | MN582486 | MN582528 | Guo et al., 2020 |
|  | *Trimerodytes percarinatus* | YBU081025/GP667 | Junlian, Sichuan, China | MN582492 | MN582535 | Guo et al., 2020 |
|  | *Trimerodytes percarinatus* | YBU081026/GP668 | Junlian, Sichuan, China | MN582493 | MN582536 | Guo et al., 2020 |
|  | *Trimerodytes percarinatus* | GP865 | Sichuan, China | MN582494 | MN582537 | Guo et al., 2020 |
|  | *Trimerodytes percarinatus* | YBU091047/GP956* | Chengdu, Sichuan, China | JQ687433 | JQ687465 | Guo et al., 2020 |
|  | *Trimerodytes percarinatus* | YBU091048/GP957* | Chengdu, Sichuan, China | MN582495 | MN582539 | Guo et al., 2020 |
|  | *Trimerodytes percarinatus* | GP1038* | Chengdu, Sichuan, China | MN582466 | MN582508 | Guo et al., 2020 |
|  | *Trimerodytes percarinatus* | YBU091096/GP1040 | Chengdu, Sichuan, China | MN582467 | MN582509 | Guo et al., 2020 |
|  | *Trimerodytes percarinatus* | GP1049 | - | MN582468 | MN582510 | Guo et al., 2020 |
|  | *Trimerodytes percarinatus* | GP 1392* | Xiushan, Chongqing, China | MN582474 | MN582516 | Guo et al., 2020 |
|  | *Trimerodytes percarinatus* | YBU13246/GP2970* | Yinjiang, Guizhou, China | MN582480 | MN582522 | Guo et al., 2020 |
|  | *Trimerodytes percarinatus* | YBU14034/GP3277* | Jinping, Yunnan, China | MN582482 | MN582524 | Guo et al., 2020 |
|  | *Trimerodytes percarinatus* | YBU14040/GP3283 | Jinping, Yunnan, China | MN582483 | MN582525 | Guo et al., 2020 |
|  | *Trimerodytes percarinatus* | YBU14203/GP3502 | Shuangbai , Yunnan, China | MN582485 | MN582527 | Guo et al., 2020 |
|  | *Trimerodytes percarinatus* | YBU16009/GP4340 | Shuangbai, Yunnan, China | MN582487 | MN582529 | Guo et al., 2020 |
|  | *Trimerodytes percarinatus* | IEBRA.2015.11 | Son La, Vietnam | MN582499 | MN582543 | Guo et al., 2020 |
|  | *Trimerodytes annularis* | GP1056 | Yizhang, Hunan, China | MN582470 | MN582512 | Guo et al., 2020 |
|  | *Trimerodytes balteatus* | YBU16106/GP4485 | Guangdong, China | MN582491 | MN582534 | Guo et al., 2020 |
|  | *Trimerodytes aequifasciatus* | GP1258 | Longzhou, Guangxi, China | MN582471 | MN582513 | Guo et al., 2020 |
|  | *Trimerodytes yapingi* | YBU15296/GP 4406 | Jingdong, Yunnan, China | MK621916 | MN582531 | Guo et al., 2020 |
|  | *Afronatrix anoscopus* | - | - | AF420073 | AF420075 | Download from NCBI |
|  | *Amphiesma craspedogaster* | - | - | GQ281781 | JQ687459 | Download from NCBI |
|  | *Amphiesma sauteri* | - | - | AF402905 | AF384824 | Download from NCBI |
|  | *Amphiesma stolatum* | - | - | JQ687432 | JQ687464 | Download from NCBI |
|  | *Atretium yunnanensis* | - | - | GQ281787 | JQ687463 | Download from NCBI |
|  | *Clonophis kirtlandii* | - | - | AF402908 | AF384827 | Download from NCBI |
|  | *Hebius parallelum* | - | - | MK201567 | MK199116 | Download from NCBI |
|  | *Hebius vibakari* | - | - | MK201296 | MK198889 | Download from NCBI |
|  | *Liodytes rigida* | - | - | AF471052 | MK198889 | Download from NCBI |
|  | *Natrix maura* | - | - | AY866530 | AY870616 | Download from NCBI |
|  | *Natrix natrix* | - | - | AF471059 | AY870640 | Download from NCBI |
|  | *Natrix tessellata* | - | - | EU119168 | AY870641 | Download from NCBI |
|  | *Nerodia erythrogaster* | - | - | GQ285504 | GQ285402 | Download from NCBI |
|  | *Nerodia fasciata* | - | - | AY866529 | AY870612 | Download from NCBI |
|  | *Nerodia harteri* | - | - | AF402935 | AF384854 | Download from NCBI |
|  | *Nerodia sipedon* | - | - | AF402913 | DQ915161 | Download from NCBI |
|  | *Nerodia taxispilota* | - | - | AF402914 | AF384833 | Download from NCBI |
|  | *Opisthotropis cheni* | - | - | GQ281779 | JQ687457 | Download from NCBI |
|  | *Opisthotropis guangxiensis* | - | - | GQ281776 | JQ687462 | Download from NCBI |
|  | *Opisthotropis lateralis* | - | - | GQ281782 | JQ687461 | Download from NCBI |
|  | *Opisthotropis maxwelli* | - | - | MK201466 | MK199024 | Download from NCBI |
|  | *Pseudoxenodon rudis* | - | - | GQ281780 | JQ687458 | Download from NCBI |
|  | *Regina alleni* | - | - | AF402916 | AF384835 | Download from NCBI |
|  | *Regina grahami* | - | - | AF402918 | AF384837 | Download from NCBI |
|  | *Regina septemvittata* | - | - | AF402917 | AF384836 | Download from NCBI |
|  | *Rhabdophis nuchalis* | - | - | AF402907 | AF384826 | Download from NCBI |
|  | *Rhabdophis tigrinus* | - | - | GQ281785 | JQ687460 | Download from NCBI |
|  | *Storeria dekayi* | - | - | AF471050 | EF417460 | Download from NCBI |
|  | *Thamnophis atratus* | - | - | AF420085 | AF420087 | Download from NCBI |
|  | *Thamnophis brachystoma* | - | - | AF420089 | HM630351 | Download from NCBI |
|  | *Thamnophis butleri* | - | - | AF402923 | HM630350 | Download from NCBI |
|  | *Thamnophis chrysocephalus* | - | - | AF420108 | AF420097 | Download from NCBI |
|  | *Thamnophis couchii* | - | - | AF402936 | AF384855 | Download from NCBI |
|  | *Thamnophis cyrtopsis* | - | - | EF417412 | EF417459 | Download from NCBI |
|  | *Thamnophis elegans* | - | - | AF402925 | AF384844 | Download from NCBI |
|  | *Thamnophis eques* | - | - | AF420117 | AF420119 | Download from NCBI |
|  | *Thamnophis exsul* | - | - | AF420125 | AF420127 | Download from NCBI |
|  | *Thamnophis fulvus* | - | - | AF420129 | AF420131 | Download from NCBI |
|  | *Thamnophis gigas* | - | - | AF420133 | AF420209 | Download from NCBI |
|  | *Thamnophis godmani* | - | - | AF420135 | AF420137 | Download from NCBI |
|  | *Thamnophis hammondii* | - | - | AF420139 | AF420141 | Download from NCBI |
|  | *Thamnophis marcianus* | - | - | AF402926 | AF384845 | Download from NCBI |
|  | *Thamnophis melanogaster* | - | - | EF417410 | EF417457 | Download from NCBI |
|  | *Thamnophis mendax* | - | - | AF420151 | AF420152 | Download from NCBI |
|  | *Thamnophis ordinoides* | - | - | AF402927 | AF384846 | Download from NCBI |
|  | *Thamnophis proximus* | - | - | AF402928 | AF384847 | Download from NCBI |
|  | *Thamnophis radix* | - | - | AF402934 | HM630344 | Download from NCBI |
|  | *Thamnophis rufipunctatus* | - | - | AF420173 | AF420175 | Download from NCBI |
|  | *Thamnophis sauritus* | - | - | AF420177 | AF420179 | Download from NCBI |
|  | *Thamnophis scalaris* | - | - | AF420181 | AF420183 | Download from NCBI |
|  | *Thamnophis scaliger* | - | - | AF420189 | AF420191 | Download from NCBI |
|  | *Thamnophis sirtalis* | - | - | AF402929 | DQ995396 | Download from NCBI |
|  | *Thamnophis sumichrasti* | - | - | AF420197 | AF420199 | Download from NCBI |
|  | *Thamnophis validus* | - | - | EF417408 | EF417437 | Download from NCBI |
|  | *Tropidoclonion lineatum* | - | - | AF420205 | AF420207 | Download from NCBI |
|  | *Virginia striatula* | - | - | AF402933 | AF384852 | Download from NCBI |
|  | *Xenochrophis flavipunctatus* | - | - | - | FJ416748 | Download from NCBI |
|  | *Xenochrophis punctulatus* | - | - | AF471079 | AY487035 | Download from NCBI |
|  | *Acrochordus granulatus* | - | - | AB177879 | - | Download from NCBI |

Abbreviations used as follows: GP, Field tag of Yibin University; YBU, Yibin University; MVZ, Museum of Vertebrate Zoology, USA; IEBR, Institute of Ecology and Biological Resources, Vietnam ; KIZ: Kunming Institute of Zoology, China; KIZ YPX, Field tag of KIZ; LJT, Field tag of Dr. J.T. Li; ROM, Royal Ontario Museum,Canada ; TBU, Tay Bac University, Vietnam; VNMN, Vietnam National Museum of Nature, Vietnam; CAS, California Academy of Sciences, USA. Specimens marked with the “*” were also used for RAD-seq. “-” indicates no data.
