# Supplementary material for: Genomic analysis reveals deep population divergence in the water snake Trimerodytes percarinatus (Serpentes, Natricidae)
Source: Ecol Evol. 2024 Apr 15;14(4):e11278. doi: 10.1002/ece3.11278 (PMC11019134; doi:10.1002/ece3.11278)
Supplement: Supplementary file 4 — Table S2. [file ECE3-14-e11278-s007.docx]

Table S2 Bioclimatic variables used in this study

| **Variable** | **Description** |
| --- | --- |
| Bio01 | Mean annual temperature |
| Bio02 | Mean diurnal range |
| Bio03 | Isothermality |
| Bio04 | Temperature seasonality |
| Bio08 | Mean temperature of wettest quarter |
| Bio10 | Mean temperature of warmest quarter |
| Bio12 | Annual precipitation |
| Bio13 | Precipitation of wettest month |
| Bio14 | Precipitation of driest month |
| Bio15 | Precipitation seasonality (coefficient of variation) |
| Bio18 | Precipitation of warmest quarter |
