# Supplementary material for: Genomic analysis reveals deep population divergence in the water snake Trimerodytes percarinatus (Serpentes, Natricidae)
Source: Ecol Evol. 2024 Apr 15;14(4):e11278. doi: 10.1002/ece3.11278 (PMC11019134; doi:10.1002/ece3.11278)
Supplement: Supplementary file 5 — Table S3. [file ECE3-14-e11278-s001.docx]

Table S3 Average sequence divergence estimates (mean uncorrected-p distances) between and within five lineages of *Trimerodytes percarinatus* defined by the mtDNA phylogeny. Interlineage distance is calculated from *cytb* (below the diagonal) and *ND2* (above the diagonal); intralineage distance is calculated from *cytb*/*ND2* (on the diagonal).

| **Lineages** | **A** | **B** | **C** | **D** | **E** |
| --- | --- | --- | --- | --- | --- |
| **A** | 0.17%/0.14% | 4.37% | 4.55% | 4.78% | 4.86% |
| **B** | 5.58% | 0.10%/0.39% | 2.77% | 3.45% | 3.18% |
| **C** | 5.59% | 3.62% | 1.25%/1.21% | 3.16% | 3.02% |
| **D** | 5.84% | 3.95% | 3.59% | 0.21%/0.32% | 2.83% |
| **E** | 6.02% | 3.98% | 3.39% | 3.50% | 1.14%/1.28% |
