# Supplementary material for: Genomic analysis reveals deep population divergence in the water snake Trimerodytes percarinatus (Serpentes, Natricidae)
Source: Ecol Evol. 2024 Apr 15;14(4):e11278. doi: 10.1002/ece3.11278 (PMC11019134; doi:10.1002/ece3.11278)
Supplement: Supplementary file 6 — Table S4. [file ECE3-14-e11278-s006.docx]

Table S4 Genetic diversity and demographic statistics of mtDNA (*cytb*/*ND2*/*cytb*+*ND2*) for each lineage

| **Lineages** | **N** | **S** | **H** | **Hd** | **π** | **K** |
| --- | --- | --- | --- | --- | --- | --- |
| **A** | 18/18/18 | 3/7/10 | 4/6/8 | 0.47/0.49/0.75 | 0.001/ 0.001/0.001 | 0.52/0.88/1.39 |
| **B** | 8/8/8 | 3/8/11 | 4/4/5 | 0.75/0.75/0.79 | 0.001/0.004/0.003 | 1.04/ 3.89/4.93 |
| **C** | 37/38/36 | 67/21/88 | 26/15/26 | 0.98/0.94/0.98 | 0.013/0.013/0.013 | 10.57/3.65/14.40 |
| **D** | 36/36/36 | 12/12/24 | 11/7/14 | 0.69/0.55/0.84 | 0.002/0.003/0.002 | 1.66/2.11/3.77 |
| **E** | 75/78/74 | 26/46/73 | 21/25/30 | 0.90/0.90/0.92 | 0.009/0.015/0.012 | 4.07/10.90/15.34 |
| **Overall** | 174/178/172 | 56/45/101 | 42/43/62 | 0.95/0.93/0.96 | 0.031/ 0.029/0.030 | 11.35/8.06/19.52 |

Abbreviations: N, number of individuals; S, number of polymorphic sites; H, number of haplotypes; Hd, haplotype diversity; π, nucleotide diversity; K, average number of pairwise differences.
