# Supplementary material for: Genomic analysis reveals deep population divergence in the water snake Trimerodytes percarinatus (Serpentes, Natricidae)
Source: Ecol Evol. 2024 Apr 15;14(4):e11278. doi: 10.1002/ece3.11278 (PMC11019134; doi:10.1002/ece3.11278)
Supplement: Supplementary file 7 — Table S5. [file ECE3-14-e11278-s003.docx]

Table S5 Results of D statistics and f4 statistics for intraspecific comparison for *T. percarinatus* lineages

| **No. of test** | **P1** | **P2** | **P3** | **O** | **D / f4 statistics** | **Std. (D/f4)** | **Z score (D/f4)** | **BABA sites** | **ABBA sites** | **nSNPs** |
| --- | --- | --- | --- | --- | --- | --- | --- | --- | --- | --- |
| **1** | Pop-I | Pop-II | Pop-III | *T. yapingi* | 0.0442 / 0.002747 | 0.004057 / 0.000253 | 10.892 / 10.841 | 9432 | 8634 | 290613 |
| **2** | Pop-I | Pop-II | Pop-IV | *T. yapingi* | -0.0498 / -0.003139 | 0.004205 / 0.000259 | -11.844 / -12.099 | 8699 | 9611 | 290613 |
| **3** | Pop-III | Pop-IV | Pop-I | *T. yapingi* | 0.0477 / 0.002876 | 0.004007 / 0.000239 | 11.915 / 12.04 | 9170 | 8334 | 290613 |
| **4** | Pop-III | Pop-IV | Pop-II | *T. yapingi* | -0.0496 / -0.00301 | 0.003715 / 0.000227 | -13.363 / -13.244 | 8373 | 9248 | 290613 |

Abbreviations: Topology for D statistics and f4 statistics is (P1, P2, P3, O), in which the O is the constant ougroup *T. yapingi* .
